# Integrative multi-omics analysis identifies SNRPE as a key driver gene in uterine corpus endometrial carcinoma: promoting tumor progression, and mediating immune evasion

**DOI:** 10.3389/fimmu.2026.1827474

**Published:** 2026-05-21

**Authors:** Minyue Cao, Yan Ding, Luyao Kang, Yiqin Zhang, Genyi Jiang, Jiayu Yan, Yihan Sun, Yanli Zhang, Jing Luo, Xue Zhou, Hanyong Wu, Bilan Li

**Affiliations:** Shanghai Key Laboratory of Maternal Fetal Medicine, Shanghai Institute of Maternal-Fetal Medicine and Gynecologic Oncology, Shanghai First Maternity and Infant Hospital, School of Medicine, Tongji University, Shanghai, China

**Keywords:** alternative splicing, immune evasion, SNRPE, T-cell exhaustion, uterine corpus endometrial carcinoma

## Abstract

**Background:**

Uterine corpus endometrial carcinoma (UCEC) has significant inherent resistance to immunotherapy. The tumor microenvironment (TME) in UCEC is characterized by limited infiltration of T cells, which has been shown to be the cause of this resistance. Recent studies have indicated that dysregulation of splicing may be involved in tumor progression and immune evasion. However, the biological mechanisms underlying this phenomenon have not been fully understood.

**Methods:**

In the study, genome-wide association studies (GWAS), splicing quantitative trait loci (sQTL), and expression quantitative trait loci (eQTL) were used for prioritizing candidate targets for complex traits and small nuclear ribonucleoprotein polypeptide E (SNRPE) was screened for the further research. The prognostic value of SNRPE was evaluated in the TCGA-UCEC cohort and validated using an independent cohort of clinical UCEC patient specimens. Its functional role was validated through *in vitro* assays and a doxycycline (Dox)-inducible xenograft model. Mechanistically, RNA sequencing (RNA-seq) was integrated with computational motif prediction and protein domain architecture analysis to identify aberrant alternative splicing (AS) events and assess their potential structural impacts. These findings were further validated via isoform-specific quantitative PCR. Concurrently, its immunomodulatory function was decoded using single-cell RNA sequencing (scRNA-seq) and experimentally validated via human peripheral blood mononuclear cells (PBMCs) co-culture assays.

**Results:**

We identified SNRPE as a driven oncogene, strongly connected to bad survival outcomes in people with UCEC. Knocking down SNRPE dramatically suppressed tumor progression both *in vitro* and *in vivo*. Mechanistically, SNRPE played a critical role in maintaining accurate splicing of dual-function hub genes (PAK1, SOS1, PIK3CB, IL17RC). Depletion of SNRPE leaded to the skipping of specific exons and alterations in protein structure, resulting in the concurrent disruption of both intrinsic oncogenic signaling pathways and tumor immune surveillance mechanisms. Meanwhile, scRNA-seq analysis unveiled that SNRPE induces an immune evasion phenotype, characterized by a specific reduction in the expression of the major histocompatibility complex class I (MHC-I) antigen presentation system and an increase of immunosuppressive midkine (MDK) signaling. Co-culture experiments confirmed that silencing SNRPE significantly restores T cell-mediated cytotoxicity and reverses the expression of exhaustion markers.

**Conclusion:**

By remodeling the global splicing network, SNRPE concurrently sustained intrinsic malignant progression and extrinsic immune suppression in UCEC.

## 1 Introduction

Uterine corpus endometrial carcinoma (UCEC), a prevalent malignancy of the female reproductive system, has demonstrated an increasing incidence trend globally (1). While the majority of patients with early-stage disease experience favorable outcomes after surgical treatment, a significant proportion (15%-20%) are either diagnosed at an advanced stage or develop postoperative recurrence (2, 3). Over the past few years, immune checkpoint blockade therapy has represented a significant advancement in the management of UCEC. Despite these promising developments, the high rates of primary resistance are an important barrier, significantly limiting the prospects for sustained clinical results in these patients (4–8). The therapeutic limitation of these results is mainly attributed to the immunosuppressed status of the tumor microenvironment (TME), characterized by the presence of hallmark features of the “cold tumor”. These characteristics are associated with limited overall T cell infiltration and an impaired cytotoxic capacity of the existing T cells, which diminishes their ability to effectively eliminate tumor cells (9, 10). Hence, the detailed understanding of the specific mechanisms underlying tumor growth promotion and the establishment of an immunosuppressive microenvironment is of considerable clinical importance.

One of the hallmarks of the development of tumors is aberrant splicing of RNA, where a single gene can produce multiple forms of mRNA through alternative splicing (AS), including the use of alternative exons, retention of introns, or splicing at alternative splice sites. This leads to increased diversity in the proteome, thereby facilitating tumorigenesis and metastasis (11–13). Beyond promoting tumor initiation and progression, splicing dysregulation has been widely recognized as a significant factor in shaping the tumor immune microenvironment, capable of generating tumor-specific neoantigens or modulating immune checkpoint signaling pathways (14–17). Although genome-wide association studies (GWAS), have identified numerous risk loci associated with UCEC susceptibility, the precise functional mechanisms by which these genetic variants influence disease progression remain unclear (18). expression quantitative trait loci (eQTL) and splicing quantitative trait loci (sQTL) provide critical bridges linking genetic variation to gene regulation, including transcriptional and post-transcriptional processes. By integrating GWAS, eQTL, and sQTL data, we can systematically prioritize splice-related genes (sGenes) with potential causal effects across multi-omics layers (19–21). Thus, through this systematic multi-omics integration strategy, it is expected to elucidate the molecular regulatory networks of diseases from a novel perspective, precisely identify sGenes with potential clinical value, and thereby provide new molecular foundations for precision diagnosis and treatment.

Through the aforementioned strategies, we have identified the core splicing factor small nuclear ribonucleoprotein polypeptide E (SNRPE) as the top candidate pathogenic gene in UCEC. As a central part of the spliceosomal Sm complex, SNRPE is essential for the formation of small nuclear ribonucleoprotein (snRNP) and for the proper process of pre-mRNA splicing (22). Although earlier researches have reported that SNRPE exhibits oncogenic properties in several solid tumors (23–26), its specific biological functions in the development and progression of UCEC has not yet been established. Notably, aberrant splicing has been widely recognized as a key factor in remodeling the TME (14–17), whether and how SNRPE, as an upstream regulator, participates in reshaping the immune microenvironment in UCEC remains unknown. Therefore, a deeper investigation of the oncogenic function of SNRPE in UCEC and its possible association with the “cold tumor” phenotype will help us re-evaluate the pathogenesis of UCEC from the perspective of RNA processing and provide a theoretical foundation for identifying novel molecular targets.

In summary, this study established a multi-omics screening framework integrating GWAS, sQTL, and eQTL data, systematically identifying SNRPE as a candidate pathogenic driver gene in UCEC. We elucidated that SNRPE may physically anchor to conserved Sm-binding motifs on pre-mRNAs to regulate the splicing pattern of dual-function hub genes. Its depletion triggers targeted exon skipping and profound protein structural defects, ultimately paralyzing both intrinsic tumor progression and extrinsic immune evasion networks.

## 2 Materials and methods

### 2.1 Multi-omics data acquisition and integrative analysis

The GWAS data for UCEC were sourced from FinnGen (https://www.finngen.fi/en), and the sQTL data were gathered from the CancerSplicingQTL database (https://cancersplicingqtl-hust.org/) (27). Furthermore, the eQTL data and transcriptomic profiles for the UCEC cohort were retrieved from the The Cancer Genome Atlas (TCGA) project via the Genomic Data Commons (GDC) data portal (https://portal.gdc.cancer.gov/). Raw count data from TCGA-UCEC were converted into transcripts per million (TPM) and normalized by applying a log_2_(TPM + 1) transformation. Samples lacking complete clinical information were excluded, yielding a final cohort of 545 UCEC samples for downstream analyzes. For comparative analysis of gene expression in normal tissues, RNA sequencing (RNA-seq) data from normal uterine tissues were obtained from the Genotype-Tissue Expression (GTEx) project (https://commonfund.nih.gov/gtex). The GTEx expression data, originally provided in TPM, were likewise log_2_(TPM + 1) transformed to ensure comparability with the TCGA dataset. After quality control, 177 normal uterine samples were retained for subsequent expression comparisons between tumor and normal groups. RNA-binding protein (RBP) binding coordinates obtained from Crosslinking and Immunoprecipitation Sequencing (CLIP-seq) experiments in the ENCODE database (https://www.encodeproject.org/) were used to investigate the mechanistic basis of splicing regulation.

### 2.2 Identification strategy for candidate sGenes

To identify candidate sGenes, this study focused the analysis on known functional loci, extracting variants from GWAS that showed suggestive associations (*p* < 0.05) with UCEC and were co-localized with functional QTLs. The genes mapped to these variants were defined as candidate sGenes. Subsequently, the enrichment of these sQTLs within RBP binding regions was analyzed using the GenomicRanges R package to explore their potential regulatory mechanisms (28).

Based on this, differentially expressed genes in UCEC were screened using the GEPIA 2.0 database (http://gepia2.cancer-pku.cn/#index) (|log2FoldChange| > 1, *p* < 0.05). To discover core splicing-related target genes, the screening results were intersected with a known set of core splicing factors (29), yielding three candidate genes: SNRPE, SNRPD2, and SNRPG. Survival data from the TCGA-UCEC cohort were further analyzed, revealing that only SNRPE exhibited stable and significant clinical prognostic value; thus, SNRPE was selected as the core target for subsequent functional validation.

### 2.3 Multidimensional feature analysis of candidate sGenes

To systematically evaluate the biological characteristics of candidate sGenes, we conducted a multidimensional integrative analysis. First, to understand their biological roles, the R package clusterProfiler was used to conduct Gene Ontology (GO) (https://geneontology.org/), Kyoto Encyclopedia of Genes and Genomes (KEGG) (https://www.genome.jp/kegg/), and Reactome enrichment studies (https://reactome.org/). Second, we analyzed somatic single nucleotide variants (SNVs) using maftools to display mutation frequencies and types for specific sGenes, and applied Spearman correlation tests to investigate associations between sGene expression and immune cell infiltration. Finally, we assessed tumor sensitivity to targeted receptor tyrosine kinase (RTK) inhibitors using data from the Genomics of Drug Sensitivity in Cancer 2 (GDSC2) database (https://www.cancerrxgene.org/). In parallel, we applied Seurat package to count an “sGene signature score” for single cells in the GSE251923 dataset (30).

### 2.4 Clinical statistical analysis and predictive modeling

Based on the median SNRPE expression, the patient cohort was stratified into high- and low-expression groups for the comparison of baseline clinicopathological characteristics. To maximize sample utilization, missing clinical data were handled using an available-case analysis approach during baseline comparisons; thus, patients were excluded only if data for the specific variable being analyzed was missing.

For subsequent prognostic evaluations, including univariate and multivariate Cox proportional hazards regression models, SNRPE expression was evaluated as a continuous variable to maximize statistical power and avoid artificial categorization bias. Prior to constructing each Cox regression model, a model-specific complete-case approach was adopted: patients with missing data in any of the adjusted clinical covariates or those lacking the specific survival endpoint of interest (including a follow-up time of zero days) were excluded solely from that specific model. Additionally, Kaplan-Meier survival curves were generated to visualize survival differences between the SNRPE-high and -low groups, with significance assessed by the Log-rank test using the survival and survminer R packages. Finally, a survival-prediction nomogram was constructed based on the multivariate Cox analysis results using the rms package.

### 2.5 Cell lines and cell culture

Human UCEC cells (Ishikawa and HEC-1B) and 293T cells were procured from the Chinese Academy of Sciences (Shanghai, China). Freshly isolated human PBMCs were cultured in RPMI 1640 medium (Gibco). The basal media for 293T and the endometrial cancer lines were DMEM (Gibco) and DMEM/F12 (Servicebio), respectively. All culture systems were enriched with 10% fetal bovine serum (FBS) and 1% penicillin-streptomycin (P/S), and cells were kept under standard conditions (37°C and 5% CO_2_).

### 2.6 Cell transfection and stable cell line generation

When the cells reached 60%-80% confluence, transient transfection was performed using siRNA targeting SNRPE with Lipofectamine RNAiMAX reagent (Thermo Fisher Scientific), followed by cell collection after 24–72 h for subsequent experiments.

To generate stable knockdown cell lines, the Tet-pLKO-puro lentiviral plasmid (YiXueSheng Biosciences) was co-transfected with packaging plasmids into 293T cells. After 48 h, viral supernatants were collected and concentrated overnight at 4°C using a concentration kit (Genomeditech). Subsequently, with the help of the infection enhancer HistransG A (Genechem), the concentrated viral particles were introduced into Ishikawa and HEC-1B cells for infection. Following infection for 48 h, cells were grown in puromycin-containing medium to undergo continuous selection over 3 days. Details of all siRNA and shRNA target sequences are provided in **Supplementary Table 1**.

### 2.7 RNA extraction, reverse transcription polymerase chain reaction (RT-PCR) and quantitative reverse transcription polymerase chain reaction (qRT-PCR)

Total RNA was isolated from cells using the RNAeasy Animal RNA Kit (Beyotime Biotechnology). RNA was then reverse-transcribed into cDNA using the HiScript III All-in-one RT SuperMix with gDNA Removal Module (Vazyme Biotech).

Subsequently, amplification and melting curve analysis were performed using SupRealQ Ultra Hunter SYBR qPCR Master Mix (Vazyme Biotech) on a QuantStudio 5 real-time fluorescent quantitative PCR instrument (Thermo Fisher Scientific). Target gene expression was quantified via the 2^-ΔΔCT^ approach, normalizing to GAPDH. Primer sequences can be found in **Supplementary Table 2**.

### 2.8 Western blotting

Cells were lysed in 1 × SDS-PAGE buffer supplemented with protease and phosphatase inhibitors (Thermo Fisher Scientific) and boiled for 10 minutes. Proteins were separated on 15% SDS-PAGE gels, transferred to PVDF membranes (Millipore), and blocked with 5% non-fat milk for 1 hour. Membranes were incubated overnight at 4°C with anti-SNRPE (1:1,000; Thermo Fisher Scientific), followed by incubation with secondary antibody (1:2,000; Proteintech). GAPDH (1:10,000; Proteintech) was used as a loading control. Protein signals were visualized using ECL reagent (Epizyme Biotech) and imaged.

### 2.9 Cell counting kit-8 (CCK-8) assay

Ishikawa and HEC-1B cells were seeded into 96-well plates at a density of 3,000 cells per well. At the designated time points, the culture medium was replaced with 100 μl of fresh medium containing 10% CCK-8 reagent (MedChemExpress) per well. The absorbance (OD) was obtained at 450 nm after 1.5 hours of incubation at 37°C in darkness.

### 2.10 Colony formation assay

The plated cells were cultured for two weeks under ideal conditions to promote colony growth. Subsequently, the colonies were fixed with 4% paraformaldehyde for 20 min to preserve cellular structure. Following fixation, they were stained with 0.5% crystal violet for another 20 min to enhance visibility. After staining, the plates were gently washed to remove excess dye, air-dried completely, and finally imaged using a high-resolution scanner for quantitative analysis.

### 2.11 5-ethynyl-2′-deoxyuridine (EdU) incorporation assay

Cells were seeded in 96-well plates for 24 h and DNA replication activity was detected using the Click-iT EdU Kit (Beyotime Biotechnology). Cells were fixed with 4% paraformaldehyde and permeabilized with 0.3% Triton X-100 and blocked with 5% BSA for 1 h. Alexa Fluor 555 labeling solution and DAPI staining solution (nuclear counterstaining) were then incubated sequentially. Images were acquired via laser microscopy (Zeiss), and EdU-positive cells were counted with ImageJ software.

### 2.12 Transwell assays

In the migration assay, Ishikawa (120,000 cells/well) or HEC-1B (30,000 cells/well) cells suspended in serum-free medium were seeded into the upper chamber. For the invasion assay, the membrane of the upper chamber was pre-coated with diluted Matrigel matrix prior to seeding higher-density cells (Ishikawa: 240,000 cells/well; HEC-1B: 60,000 cells/well). Medium containing 10% FBS was added to the lower compartments. After incubating for 20 hours, the unmigrated cells on the top surface were gently wiped away with cotton swabs. Cells that had penetrated through the membrane were sequentially fixed with 4% paraformaldehyde and stained with 0.1% crystal violet, followed by washing, drying, and photography under an optical microscope from three randomly selected fields.

### 2.13 Subcutaneous xenograft model and *in vivo* Dox induction

5-week-old female BALB/c nude mice were purchased from Shanghai Slake Laboratory Animal Co., Ltd. All animal experiments complied with the NIH Guide for the Care and Use of Laboratory Animals and were approved by the Animal Ethics Committee of Shanghai First Maternity and Infant Hospital (approval No. TJBG04823101). A total of 2 × 10 (6) HEC-1B cells stably transfected with the Tet-on-shSNRPE plasmid were subcutaneously injected into the right abdominal flank of nude mice. 7 days after injection, mice were randomly divided into two groups (n = 5 per group) and administered either 5% sucrose water (control group) or 5% sucrose water containing 1.75 mg/mL Dox (Aladdin) to induce SNRPE silencing. Drug-containing drinking water was stored in opaque bottles and replaced every 48 h. Tumor length (L) and width (W) were assessed every three days, and tumor volume (V) was computed using the formula V = (L × W (2))/2.

### 2.14 Hematoxylin and eosin staining (HE) and immunohistochemistry (IHC) staining

For HE staining, standard protocols were followed to assess tumor morphology. For IHC, tumor sections underwent antigen retrieval in citrate buffer. Following the quenching of endogenous peroxidase and blocking of non-specific binding, the tissues were then probed overnight at 4°C with primary antibodies against SNRPE (1:200; Thermo Fisher Scientific) or Ki-67 (1:500; Proteintech). HRP-conjugated secondary antibodies were applied, followed by DAB staining using a DAB kit (Vector Laboratories). Finally, sections were counterstained with hematoxylin and analyzed under a microscope.

### 2.15 Bulk RNA-seq data analysis and characterization of immune microenvironment characterization

Samples from the TCGA-UCEC cohort were classified into two groups relying on the median SNRPE expression level. Raw count data were normalized with the DESeq2 package, and differentially expressed genes (DEGs) were identified with the criteria |log2FoldChange| > 1 and FDR < 0.05. Pathway enrichment analyzes of DEGs were performed using the clusterProfiler package based on GO, KEGG, and Hallmark gene sets. To characterize the tumor immune microenvironment, the IOBR package integrated multiple deconvolution algorithms (CIBERSORT, EPIC, xCell, MCP-counter, and QuanTIseq) to estimate the relative abundance of various immune cell types. Additionally, the ESTIMATE algorithm was applied to calculate immune scores, stromal scores, and tumor purity, providing a comprehensive view of the tumor stromal landscape.

### 2.16 Single-cell RNA sequencing (scRNA-seq) analysis

Data from normal endometrium and UCEC tissues were obtained from the GEO datasets (GSE139555 (31), GSE173682 (32), and GSE179640 (33)) (https://www.ncbi.nlm.nih.gov/geo/). Quality control excluded low-quality cells using the following criteria: detected genes < 500 or > 8,000, mitochondrial gene proportion > 20%, ribosomal gene proportion < 3%, and hemoglobin gene proportion > 1%. DecontX was applied to remove ambient RNA contamination. Sample integration and correction for batch effects were carried out using the Seurat package in conjunction with the Harmony algorithm, utilizing the first 15 principal components as input for dimensionality reduction and clustering (34, 35). FindClusters and UMAP were the algorithms that were used in order to accomplish the tasks of cell clustering and dimensionality reduction. Cell populations were identified and annotated based on the expression of well-known canonical marker genes. T and NK cells were further extracted and subjected to secondary clustering to identify CD8^+^ effector memory (Tem) and exhausted (Tex) T cell subsets (36, 37). CellChat was used to reconstruct cell-cell communication networks, enabling analysis of dynamic interactions between high- and low-SNRPE groups (38). Additionally, the scMetabolism package, using KEGG gene sets, quantified metabolic pathway activities at the single-cell level.

### 2.17 Peripheral blood mononuclear cells (PBMCs) isolation and co-culture assay

Peripheral blood samples were collected from fasting healthy volunteers in EDTA tubes with approval from the Ethics Committee of Shanghai First Maternity and Infant Health Hospital (Approval No. KS23107), and informed consent was obtained from all participants. PBMCs were isolated using Ficoll-Paque density gradient centrifugation (GE Healthcare), washed, and cultured in RPMI 1640 medium with CD3/CD28 activation beads at a 1:1 ratio (Thermo Fisher Scientific) and IL-2 (BioLegend). Fresh IL-2-containing medium was added regularly. Activation beads were removed before co-culture or flow cytometry using a magnetic rack. We co-cultured activated PBMCs with UCEC cells in 96-well plates for functional assays.

### 2.18 Lactate dehydrogenase (LDH) cytotoxicity assay

The LDH cytotoxicity assay kit (Beyotime Biotechnology) was used to evaluate T cell-mediated killing. Effector and target cells were co-cultured for 48 h. Supernatants were mixed with LDH working reagent in the dark for 30 min, and absorbance was measured at 490 nm (test) and 680 nm (background). Net OD values were calculated as OD490 - OD680. Controls included medium alone, spontaneous target release (Low control), maximum target release (High control, 2% Triton X-100), and effector cell control. Specific cytotoxicity (%) was calculated as: Cytotoxicity (%) = (Effector/target cell mix -Effector cell control-Low control)/(High control - Low control) × 100.

### 2.19 Flow cytometry analysis

After co-culture, PBMCs were harvested to assess T cell phenotype and function. For activation and intracellular cytokine detection, cells were stimulated with PMA (Sigma-Aldrich) and ionomycin (MedChemExpress) in the presence of GolgiStop (BD Biosciences) for 2-3 h, with CD107a staining to evaluate degranulation. Dead cells were excluded using Fixable Viability Dye eFluor 450 (Thermo Fisher Scientific), followed by surface staining for CD45, CD3, and CD8. After fixation and permeabilization (BD Biosciences), intracellular staining for IFN-γ and TNF-α was performed. For exhaustion markers, cells collected on day 5 were directly stained for PD-1, BTLA, and other surface markers without fixation/permeabilization. All antibodies were from BioLegend unless noted. Samples were acquired on a BD flow cytometer and analyzed using FlowJo v10.9.

### 2.20 AS Validation and percent spliced in (PSI) quantification

To validate the identified exon skipping (ES) events, two complementary primer systems were designed. Flanking primers were anchored within the constitutive exons adjacent to the target exon to distinguish between inclusion and skipping isoforms based on amplicon length differences. Junction-spanning primers were specifically designed across the splicing junctions to achieve precise quantification of inclusion and skipping transcripts. Specifically, isoform-specific junction-spanning primers were utilized for qPCR to determine the relative abundance of each transcript. The PSI was calculated using the formula: PSI = Inclusion/(Inclusion + Skipping) × 100%. Isoform levels were normalized to GAPDH via the 2^-ΔΔCT^ method, following the protocols described in Section 3.7. In parallel, semi-quantitative RT-PCR was performed using EmeraldAmp PCR Master Mix (Takara Bio). The resulting amplicons were separated by 2.0% agarose gel electrophoresis, and band intensities were quantified using ImageJ software to assess the distribution of splicing isoforms. Detailed primer sequences are provided in **Supplementary Table 2**.

### 2.21 Binding motif prediction

Potential SNRPE binding sites within target pre-mRNAs were predicted using the RBPmap (http://rbpmap.technion.ac.il/) web server. Input sequences comprised the skipped exons along with their 200-bp flanking intronic regions. For the analysis, the background was set to “Human,” the stringency level was adjusted to “High,” and the “Conservation filter” was enabled. This pipeline was employed to prioritize canonical Sm-binding motifs and their physiological variants that exhibit evolutionary conservation across multiple vertebrate species.

### 2.22 Protein domain analysis

Protein functional domains of the affected genes were systematically annotated using the Ensembl database (https://www.ensembl.org/). Based on the genomic coordinates of ES events, nucleotide deletions were mapped to the corresponding protein primary structures using reference transcript sequences retrieved from the NCBI database. This allowed for a precise assessment of how splicing variations compromised the integrity of core functional modules.

### 2.23 Patient cohort and tissue microarray (TMA) analysis

TMAs were constructed from formalin-fixed paraffin-embedded (FFPE) specimens of 140 UCEC patients who underwent surgical resection at Shanghai First Maternity and Infant Hospital (Approval No. KS23107). This study was approved by the Institutional Ethics Committee, and informed consent was obtained from all participants. Clinical proliferation data (Ki67 index) for each core were retrospectively retrieved from the patients’ official diagnostic pathology reports.

SNRPE expression and CD8^+^ T cell infiltration were evaluated via IHC and immunofluorescence (IF) staining on adjacent serial TMA sections. For IHC, slides were incubated with an anti-SNRPE primary antibody (1:200; Thermo Fisher Scientific) and independently scored by two blinded pathologists to stratify the cohort into SNRPE-high (n = 70) and SNRPE-low (n = 70) groups. For IF, adjacent sections were stained with an anti-CD8 antibody (1:200; Cell Signaling Technology) and counterstained with DAPI. Images were captured via fluorescence microscopy, and the percentage of CD8^+^ cells relative to total cells was quantified using QuPath software.

### 2.24 Statistical analysis

All bioinformatics and statistical analyzes were performed using R (v4.0.3) and GraphPad Prism 9.0. *In vitro* experiments were repeated at least three times independently, and results are presented as mean ± standard deviation (mean ± SD). Comparisons between two groups were made using unpaired Student’s t-test, while multiple group comparisons were analyzed by one-way ANOVA followed by Tukey’s *post hoc* test. *In vivo* xenograft tumor growth curves were compared using two-way ANOVA. A *p*-value < 0.05 was considered statistically significant (*p < 0.05, **p < 0.01, ***p < 0.001, ****p < 0.0001).

All reagents mentioned in the Methods section are listed in **Supplementary Table 3**.

## 3 Results

### 3.1 Integrative multi-omics analysis revealed SNRPE as a key splicing-associated gene in UCEC

**Figure 1:**
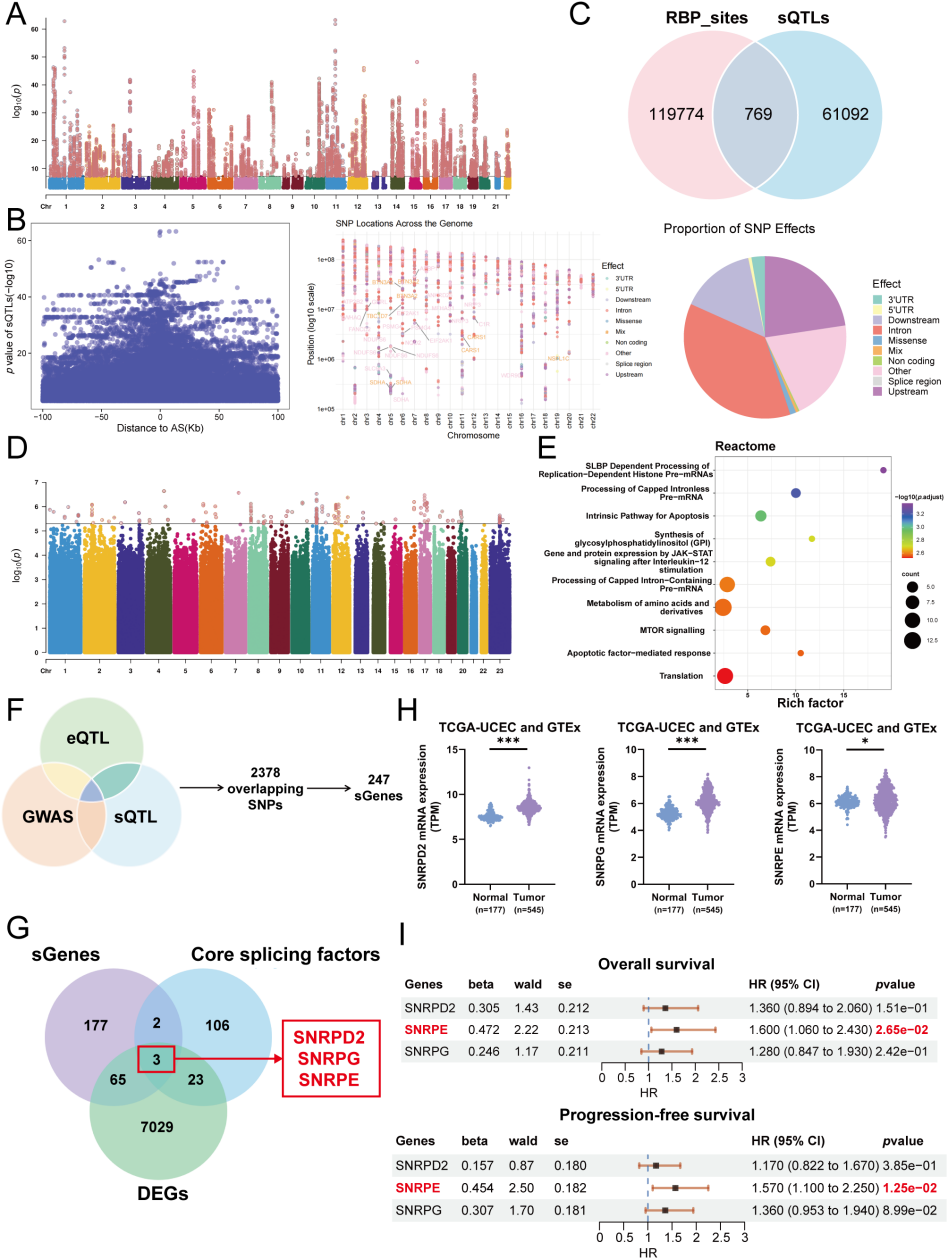
Integrative multi-omics analysis revealed SNRPE as a key splicing-associated gene in UCEC. **(A)** Manhattan plot visualizing genome-wide sQTL signals. **(B)** Functional and positional characterization of sQTLs. Left: distribution of distances between SNPs and splicing events. Middle: chromosomal distribution of sQTLs. Right: distribution of SNP functional annotations. **(C)** Venn diagram showing the overlap between sQTLs and RBP binding sites. **(D)** Manhattan plot of endometrial cancer GWAS summary statistics. **(E)** Venn diagram illustrating the identification of 247 sGenes by intersecting sQTL, eQTL, and GWAS datasets. **(F)** Reactome pathway enrichment analysis of the sGenes. **(G)** Venn diagram showing the intersection of sGenes, core splicing factors, and UCEC DEGs, identifying three candidates: SNRPE, SNRPD2, and SNRPG. **(H)** The expression of the three candidate genes in normal and tumor tissues. **(I)** Forest plot summarizing the prognostic value (hazard ratio (HR) for OS and PFS) of the three candidates. *p < 0.05, ***p < 0.001.

In order to understand the background of UCEC, we conducted a genome-wide sQTL association analysis by integrating paired genotypic and transcriptomic data. The analysis revealed splice-altering single nucleotide polymorphisms (SNPs) across the genome. The SNPs were found to be enriched in intronic regions as well as in close proximity to canonical splice sites, indicating a strong association with key genomic regulatory elements involved in splicing (**Figures 1A, B**). Interestingly, a significant percentage of these sQTLs were found to be in regions bound by RBPs, indicating their putative role in splicing regulation (**Figure 1C**). Subsequently, we conducted a cross-integration of these SNPs with eQTL and UCEC GWAS data (**Figure 1D**) to reveal high-confidence pathogenic variants that were simultaneously associated with splicing dysregulation, gene expression, and disease susceptibility. This integrative analysis narrowed down the list of candidate targets to only 247 sGenes (**Figure 1E**).

The sGenes were found to be enriched in RNA metabolism, including pre-mRNA splicing, as well as in key oncogenic signaling pathways, such as mTOR and apoptosis (**Figure 1F**). These results showed that the identified genes may significantly contribute to tumor progression via post-transcriptional regulatory mechanisms. Subsequently, a comprehensive characterization of these sGenes was conducted from genomic, immunological microenvironmental, and pharmacological viewpoints. Mutation analysis showed that SETX exhibited the greatest mutation frequency among the genes within this set (**Supplementary Figures 1A, B**). At the level of immunological microenvironment, there was a strong correlation between sGene expression and the infiltration of multiple immune cells (**Supplementary Figures 1C, D**). Pharmacogenomic analysis further uncovered potential associations between sGenes and sensitivity to RTK-targeted therapeutics (**Supplementary Figures 1E, F**). Additionally, scRNA-seq data demonstrated that sGenes are highly expressed in malignant epithelial cells and T cells (**Supplementary Figure 1G**).

To further identify core target genes with clinical translational potential, we performed a cross-validation analysis among sGenes, the known list of core splicing factors (29), and DEGs from the UCEC cohort in the GEPIA 2.0 database (**Supplementary Table 4**). This integrative approach revealed three overlapping candidate genes which included SNRPE, small nuclear ribonucleoprotein D2 (SNRPD2), and small nuclear ribonucleoprotein G (SNRPG) (**Figure 1G**). Based on TCGA and GTEx analyzes, UCEC tissues exhibited substantially increased levels of the three genes relative to normal tissues (**Figure 1H**). However, further survival analysis revealed that only SNRPE exhibited stable prognostic value which was consistently and strongly associated with diminished overall survival (OS) and progression-free survival (PFS), whereas SNRPD2 and SNRPG showed inconsistent prognostic trends (**Figure 1I**). Integrating genetic colocalization, differential expression, and prognostic correlation analyzes, SNRPE was identified as the central target gene for subsequent functional experiments and mechanistic investigations in this study.

### 3.2 SNRPE as an independent prognostic marker and tool for clinical prediction in UCEC

**Figure 2:**
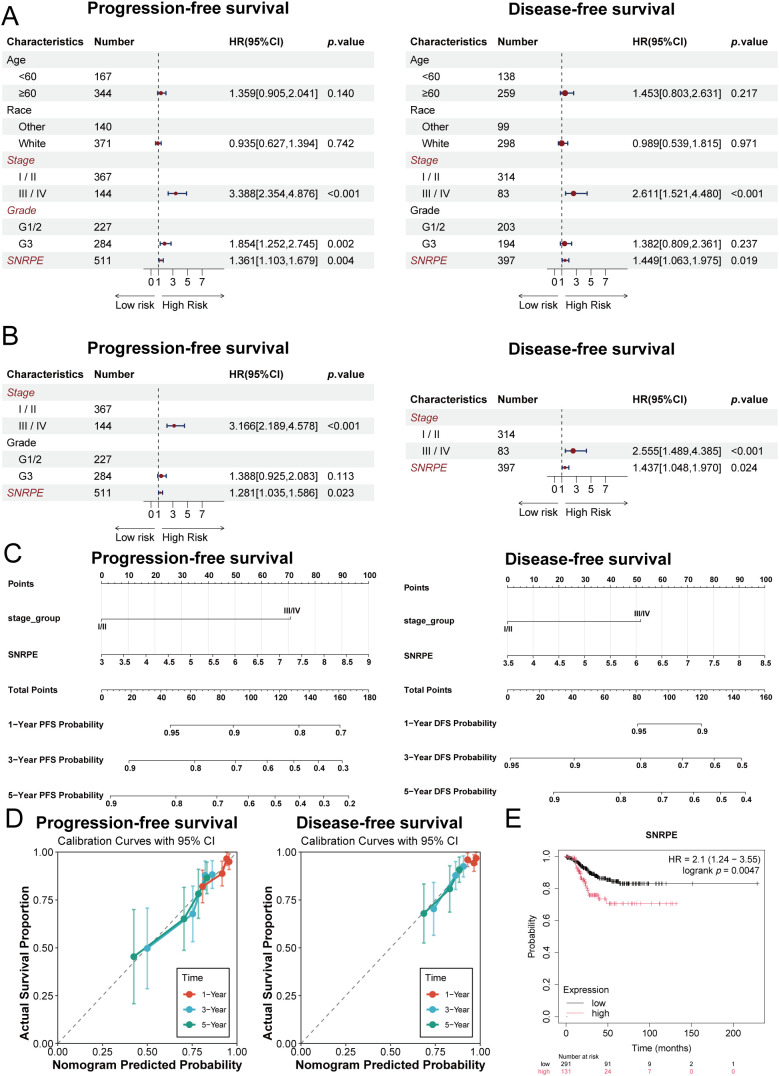
SNRPE as an independent prognostic marker and tool for clinical prediction in UCEC. **(A)** Forest plots of univariate Cox proportional hazards regression analysis for SNRPE expression and survival outcomes (PFS and DFS). **(B)** Forest plots of multivariate Cox regression analysis for PFS and DFS adjusted for confounding clinical variables. **(C)** Prognostic nomograms integrating SNRPE expression with tumor stage and histological grade for 1-, 3-, and 5-year PFS and DFS. **(D)** Calibration curves comparing predicted survival probabilities with observed clinical outcomes. The diagonal line represents ideal prediction. **(E)** Kaplan–Meier survival curves of RFS from the Kaplan-Meier Plotter dataset stratified by SNRPE expression levels.

Following this, we conducted a systematic assessment of the clinical prognostic value of SNRPE in UCEC. First, univariate Cox regression analysis indicated that the higher the expression level of SNRPE, the worse the prognosis, including reduced PFS, lower disease-free survival (DFS) (**Figure 2A**), and decreased disease-specific survival (DSS) (**Supplementary Figure 2A**). Although the association with OS was relatively weak in univariate analysis (**Supplementary Figure 2B**), multivariate Cox regression, adjusted for potential confounding variables, confirmed that SNRPE remained an independent risk factor for PFS and DFS (*p* < 0.05, HR > 1) (**Figure 2B**). Furthermore, Kaplan-Meier analysis demonstrated that high SNRPE expression was also associated with poorer recurrence-free survival (RFS) (**Figure 2E**). Finally, analysis of baseline clinical characteristics revealed that high SNRPE expression was significantly associated with advanced histological grades, older age, and non-white ethnicity (**Table 1**).

**Table 1:** Comparison of clinical characteristics between low and high SNRPE expression groups in UCEC patients.

Clinical characteristic	Low SNRPE expression (n = 273)	High SNRPE expression (n = 272)	*p* value
Age (years)			
< 60	104 (38.1%)	75 (27.6%)	0.012
≥ 60	168 (61.9%)	197 (72.4%)	
Stage			
I	171 (62.6%)	169 (62.1%)	0.750
II	24 (8.8%)	28 (10.3%)	
III	61 (22.3%)	63 (23.2%)	
IV	17 (6.2%)	12 (4.4%)	
Grade			
G1/2	145 (53.1%)	86 (31.6%)	<0.001
G3	128 (46.9%)	186 (68.4%)	
Race			
White	204 (78.5%)	169 (66.8%)	0.004
Others	56 (21.5%)	84 (33.2%)	

On the basis of these findings, we constructed a prognostic model to predict the 1-, 3-, and 5-year survival rate of UCEC patients (**Figure 2C**). The model has high predictive accuracy, according to the calibration curves (**Figure 2D**). Moreover, the receiver operating characteristic (ROC) analysis of the OS of the patients using the time-dependent ROC curve was carried out. It turned out that the model’s area under the curve (AUC) values of 1-, 3-, and 5-year were 0.556, 0.587, and 0.635 (**Supplementary Figure 2C**). This shows that the model’s high predictive accuracy, making the findings produced from it trustworthy.

### 3.3 SNRPE is a major driving force for the malignant transformation of UCEC *in vitro* and *in vivo*

**Figure 3:**
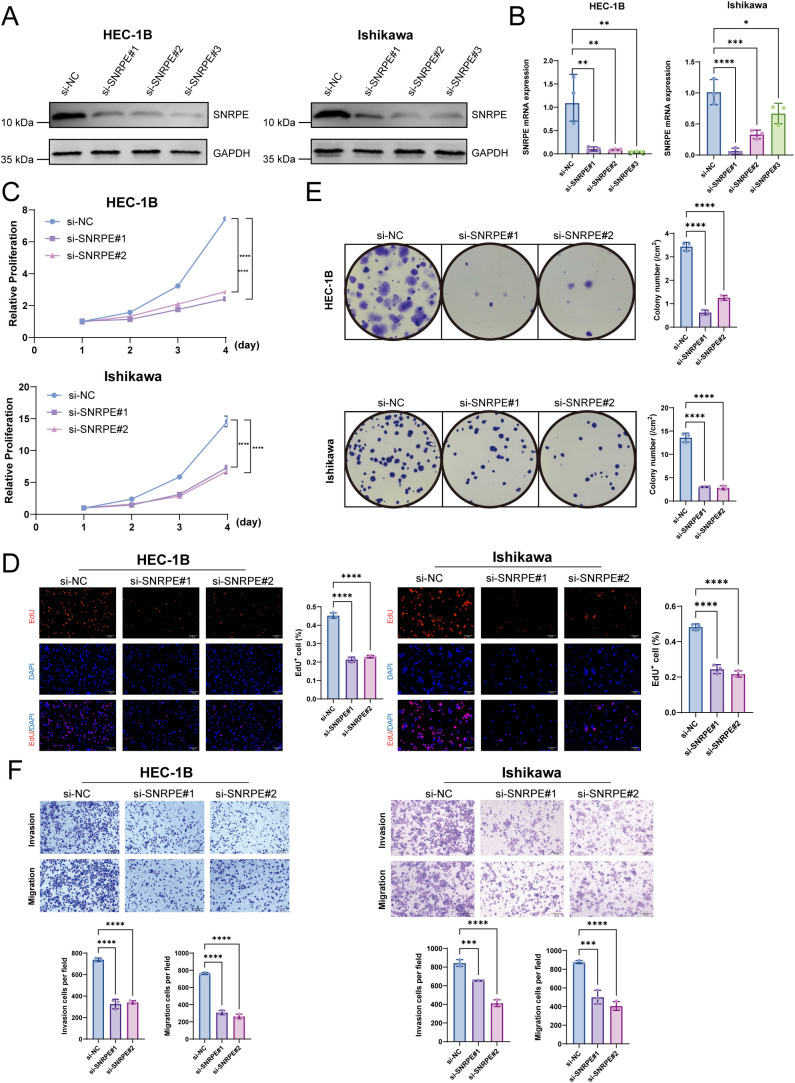
SNRPE depletion inhibits UCEC proliferation and metastasis *in vitro*. **(A, B)** Validation of SNRPE knockdown efficiency. Western blot **(A)** and qPCR **(B)** analyzes of SNRPE expression in HEC-1B and Ishikawa cells transfected with specific siRNAs. **(C)** Cell viability measured by CCK-8 assay. **(D)** DNA synthesis measured by EdU incorporation assay. **(E)** Clonogenic capacity measured by colony formation assay. **(F)** Transwell assays assessing migration (bottom) and invasion (top) capabilities. Scale bar = 200 μm. *p < 0.05, **p < 0.01, ***p < 0.001, ****p < 0.0001.

Firstly, three specific siRNAs were transfected into the representative endometrioid adenocarcinoma cell lines (HEC-1B and Ishikawa) to examine the function of SNRPE in the malignant transformation of UCEC. Western blot and quantitative PCR (qPCR) analysis were performed and confirmed the efficient knockdown of SNRPE at the protein and mRNA levels, respectively (**Figures 3A, B**). Accordingly, si-SNRPE#1 and si-SNRPE#2 were selected for further studies. The findings indicated that the suppression of SNRPE markedly suppressed the proliferation of tumor cells, as evidenced through the CCK-8, EdU, and colony formation assays (**Figures 3C–E**). Moreover, the Transwell assays showed that the knockdown of SNRPE significantly inhibited the migratory and invasive ability of the cancer cells (**Figure 3F**).

**Figure 4:**
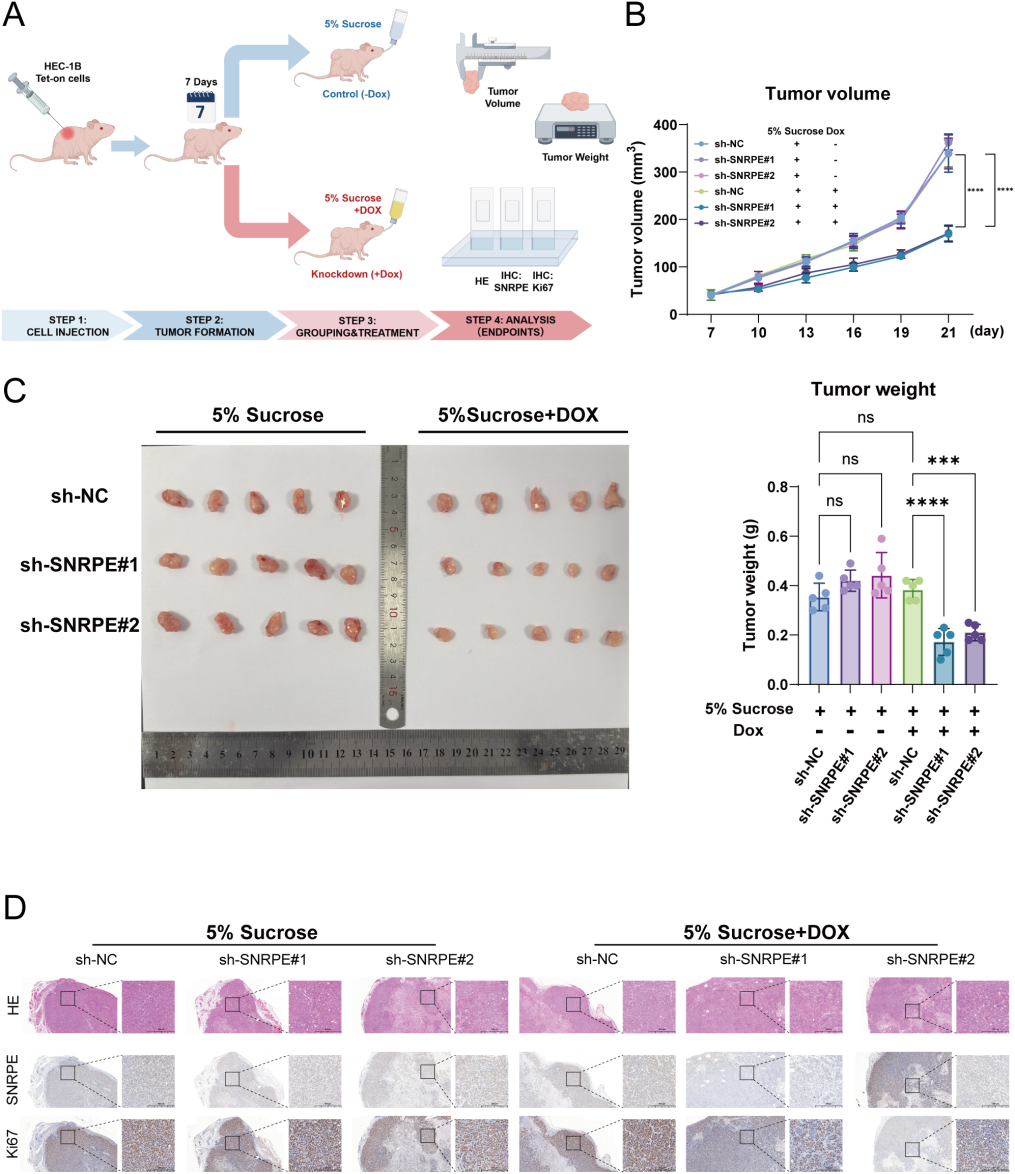
Dox-inducible knockdown of SNRPE suppresses tumorigenesis *in vivo*. **(A)** Experimental design schematic. By Figdraw. **(B)** Tumor growth curves monitored over time. **(C)** Comparison of final tumor weights between the control (-Dox) and knockdown (+Dox) groups at the endpoint. **(D)** Representative histological images of xenograft tumors. Top: HE staining showing tumor architecture. Middle: IHC staining of SNRPE. Bottom: IHC staining of Ki-67. Scale bar = 500 μm. ***p < 0.001, ****p < 0.0001.

To address the constraints of transient transfection and the possible side effects of siRNA, the Dox-inducible stable knockdown model Tet-on-shSNRPE was constructed. The results showed that the stable knockdown of SNRPE *in vitro* only occurred in the presence of Dox and significantly inhibited the malignant phenotype of the tumor cells through the CCK-8, EdU, Colony formation, and Transwell assays, respectively (**Supplementary Figures 3A–E**). The *in vivo* xenograft models were generated by injecting the HEC-1B Tet-on-shSNRPE cells into the mice. The results showed that the inducible stable knockdown of SNRPE significantly inhibited the growth of the tumor, as evidenced by the significantly smaller tumor volume and weight in the stable knockdown group compared with the controls at the endpoint of the experiment (**Figures 4A–C**). The HE staining showed the presence of well-defined solid tumor tissue, and the IHC staining revealed the absence of nuclear SNRPE and the presence of reduced Ki-67 in the tumor tissue of the stable knockdown group (**Figure 4D**).

### 3.4 SNRPE remodels the immunosuppressive microenvironment promoting CD8^+^ T cell exhaustion and dysfunction

**Figure 5:**
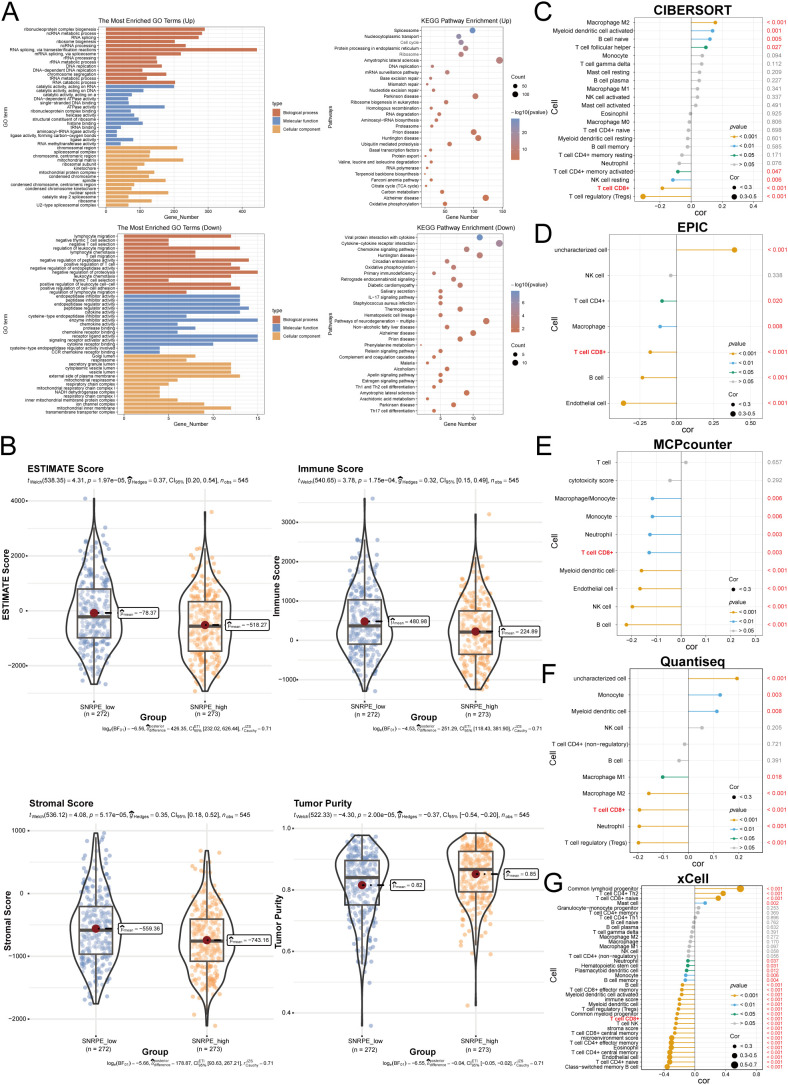
Multi-algorithmic analysis reveals decreased CD8^+^ T-cell abundance in SNRPE-high tumors. **(A)** Functional enrichment analyzes of DEGs. Top panels: GO (left) and KEGG (right) enrichment of upregulated genes. Bottom panels: GO (left) and KEGG (right) enrichment of downregulated genes. **(B)** ESTIMATE analysis comparing tumor purity, immune score, and stromal score between SNRPE-high and SNRPE-low groups. **(C–G)** Lollipop charts visualizing the correlation between SNRPE expression and immune cell infiltration levels estimated by five independent algorithms: **(C)** CIBERSORT; **(D)** EPIC; **(E)** MCPcounter; **(F)** QuanTIseq; and **(G)** xCell.

To elucidate the molecular mechanisms associated with SNRPE in the UCEC progression, we conducted differential gene expression analysis using transcriptomic data from TCGA database. The analysis revealed notable transcriptional distinctions between two groups (**Supplementary Figures 4A, B**). Functional enrichment analysis revealed a key immunomodulatory feature: up-regulated genes were predominantly involved in spliceosomes and cell cycle pathways, while down-regulated genes were highly focused on immune cell recruitment and activation (**Figure 5A**). Specifically, GO and KEGG enrichment results collectively point to inhibition of lymphocyte migration and inhibition of cytokine receptor interaction signals. The ESTIMATE algorithm further confirmed that high SNRPE expression was found to correlate with tumor purity and significantly reduced immune score (**Figure 5B**), suggesting that SNRPE may be involved in the construction of an immunosuppressive microenvironment.

To further explore the specific cell subsets affected in the TME, five independent algorithms for the analysis of immune cell infiltration were integrated. The results showed that all algorithms identified that high levels of SNRPE were significantly correlated with decreased CD8^+^ T cell abundance (**Figures 5C–G**). Notably, the xCell algorithm showed that although the proportion of CD8^+^ T cells was slightly increased, the functional subsets of effector memory CD8^+^ T cells (CD8 Tem) and central memory CD8^+^ T cells (CD8 Tcm) were significantly excluded from tumor tissues (**Figure 5G**). These results indicate that functional CD8^+^ T cell proportion in the TME may be inhibited by high SNRPE levels.

**Figure 6:**
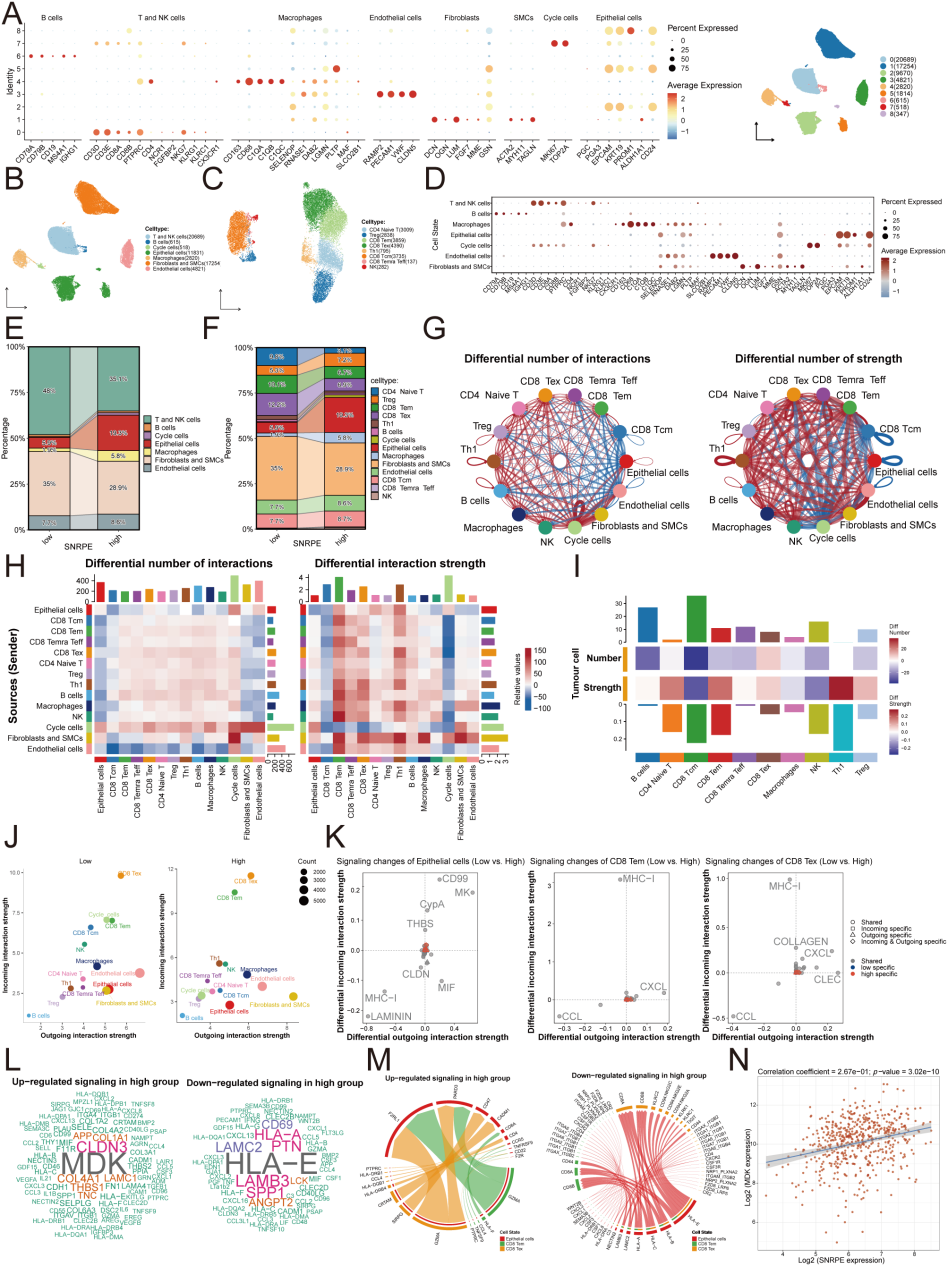
Single-cell atlas reveals SNRPE-mediated T-cell exhaustion and rewiring of cell-cell communication networks. **(A)** UMAP presentation of 7 main cell clusters identified from the scRNA-seq cohort. **(B)** Dot plot showing the level of canonical marker genes used to annotate the major cell types: T and NK cells, B cells, proliferating cells (cycle cell), epithelial cells, macrophages, fibroblasts and smooth muscle cells (fibroblasts and SMCs), and endothelial cells. **(C)** Re-clustering of T and NK cells. UMAP plot showing the further stratification of the initial T/NK cluster into 8 distinct subtypes: CD4^+^ naive T cells, regulatory T cells (Treg), CD8^+^ effector memory T cells (CD8 Tem), CD8^+^ exhausted T cells (CD8 Tex), T helper type 1 cells (Th1), CD8^+^ central memory T cells (CD8 Tcm), CD8^+^ terminally differentiated effector memory T cells (CD8 Temra Teff), and natural killer cells (NK). **(D)** Dot plot displaying the canonical markers applied to identify distinct T-cell sub-clusters. Stacked bar plots illustrating the cellular composition in SNRPE-low and SNRPE-high groups **(E, F)**. Circle plots **(G)**, heatmap **(H)**, and bar plots **(I)** visualizing changes in cell-cell interactions. Red indicates increased interaction strength in the SNRPE-high group; blue indicates decreased strength. **(J)** Scatter plots showing the dominant incoming (y-axis) and outgoing (x-axis) signaling roles. **(K)** Differential signaling pathway analysis. Scatter plot identifying specific signaling changes between groups. Positive values indicate signaling pathways upregulated in the SNRPE-high group, whereas negative values indicate pathways upregulated in the SNRPE-low group. **(L)** Word cloud visualization of significantly altered signaling pathways (Left: upregulated; Right: downregulated). **(M)** Chord diagrams of ligand-receptor interactions (Left: upregulated; Right: downregulated). **(N)** Scatter plot illustrating the positive correlation between SNRPE and MDK mRNA expression levels in the TCGA-UCEC dataset (R = 0.512).

To further explore the mechanisms of the immunosuppressive status in the TME, the scRNA-seq data from public databases were used for analysis. First, seven major cell types were identified, and the T and natural killer (NK) cells subsets were further divided into eight subsets (**Figures 6A–D**). The results showed that the proportion of cytotoxic CD8^+^ T cells, including effector memory CD8^+^ T cells (CD8 Tem) and exhausted CD8^+^ T cells (CD8 Tex), was dramatically reduced in the tumor with high levels of SNRPE expression (**Figures 6E, F**). Notably, the CellChat algorithm showed that although the proportion of CD8^+^ T cells was reduced in the tumor tissues with high levels of SNRPE expression, the interaction strength between tumor cells and CD8^+^ T cells was significantly increased in the high SNRPE context (**Figures 6G–J**).

Further molecular analysis of these interactions revealed a key regulatory pathway involved in this transition from “immune recognition” to “immune suppression” (**Figures 6K–M**). Specifically, there was an interruption in the transmission of antigens to the TME because of a breakdown in the signaling between major histocompatibility complex class I (MHC-I) molecules and the receptors for these molecules. At the same time, there was upregulation of pro-tumor activity, as revealed by changes in midkine (MDK) signaling (**Figure 6L**). MDK is a potent inhibitory ligand that can directly inhibit T cell activation, indicating that this transition is shifting the immune microenvironment from one of immune surveillance to one of immune suppression. Moreover, we also found a positive correlation between SNRPE and MDK expression in the TCGA database (**Figure 6N**).

**Figure 7:**
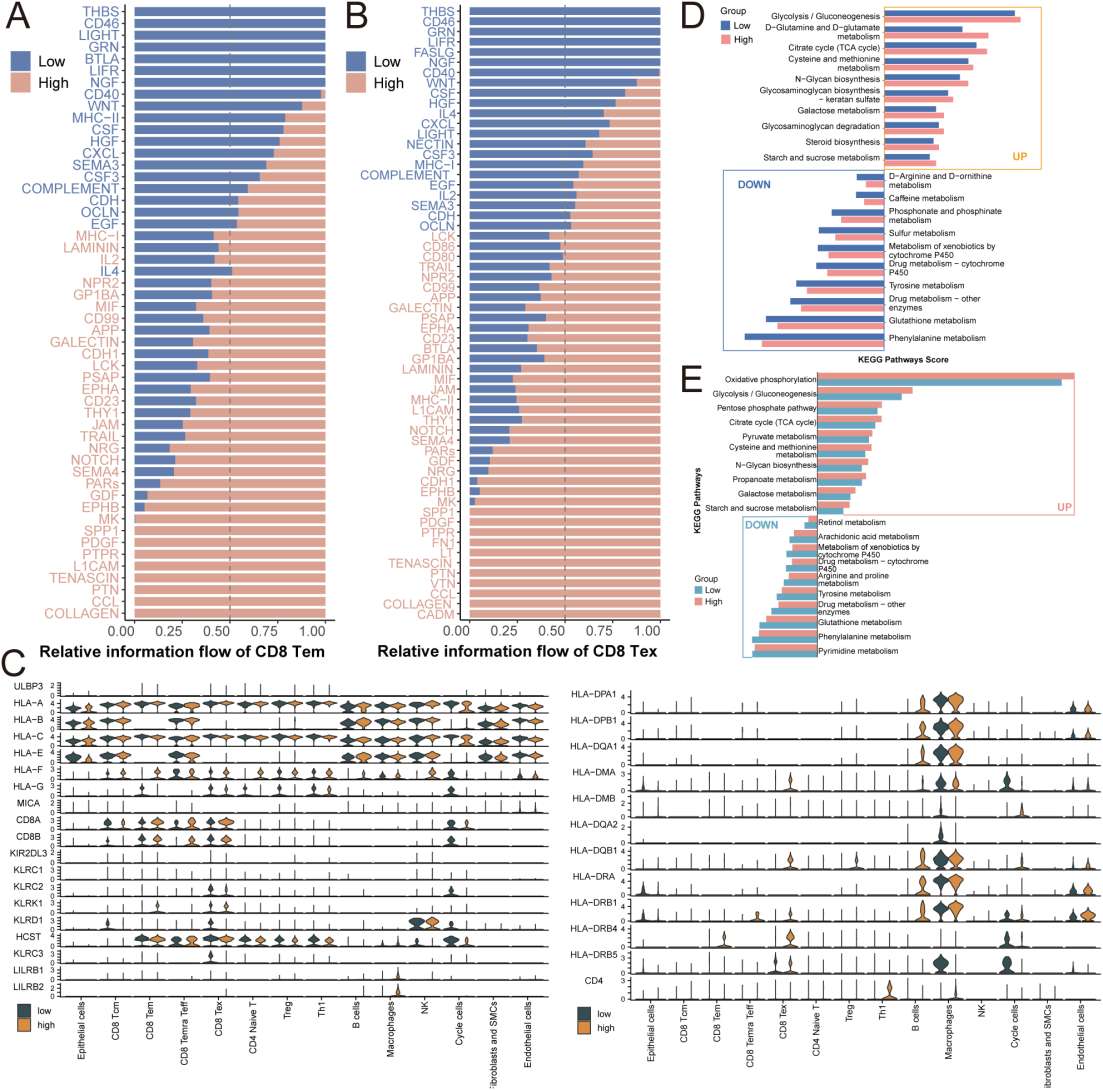
SNRPE promotes immune evasion via MHC-I downregulation and T-cell metabolic reprogramming. Bar plots showing the signaling pathways dominant in SNRPE-low (blue) or SNRPE-high (pink) groups for **(A)** CD8 Tem and **(B)** CD8 Tex cells. **(C)** Violin plots of gene expression. Comparison of pathway enrichment scores across different pathways in CD8 Tem **(D)** and CD8 Tex **(E)** cells is shown as bar charts.

To further understand how these altered interactions impact T-cell states, we analyzed the relative information flow of signaling pathways. We observed that the dominant signaling patterns governing CD8^+^ Tem and Tex cells were distinct between high and low SNRPE conditions, with a notable shift in the prominence of immune-modulatory pathways (**Figures 7A, B**). The transcriptomic analysis revealed further evidence of immune evasion by these cancer cells. The malignant epithelial cancer cells that exhibited high SNRPE expression exhibited downregulation of MHC-I and MHC-II gene expression (**Figure 7C**), indicating reduced tumor immunogenicity, including reduced recognition by cytotoxic T cells. At the same time, there was pronounced metabolic reprogramming of CD8^+^ T cells. The KEGG pathway analysis revealed that the metabolic changes of CD8 Tem (**Figure 7D**) and CD8 Tex cells (**Figure 7E**) in the different groups of SNRPE expression. In the high SNRPE-expressing environment, these cells exhibited hyperactive central carbon metabolism, including glycolysis and oxidative phosphorylation. The hyperactive metabolism is characteristic of chronic T cell exhaustion (39–41), indicating that SNRPE could be accelerating this exhaustion response in T cells.

**Figure 8:**
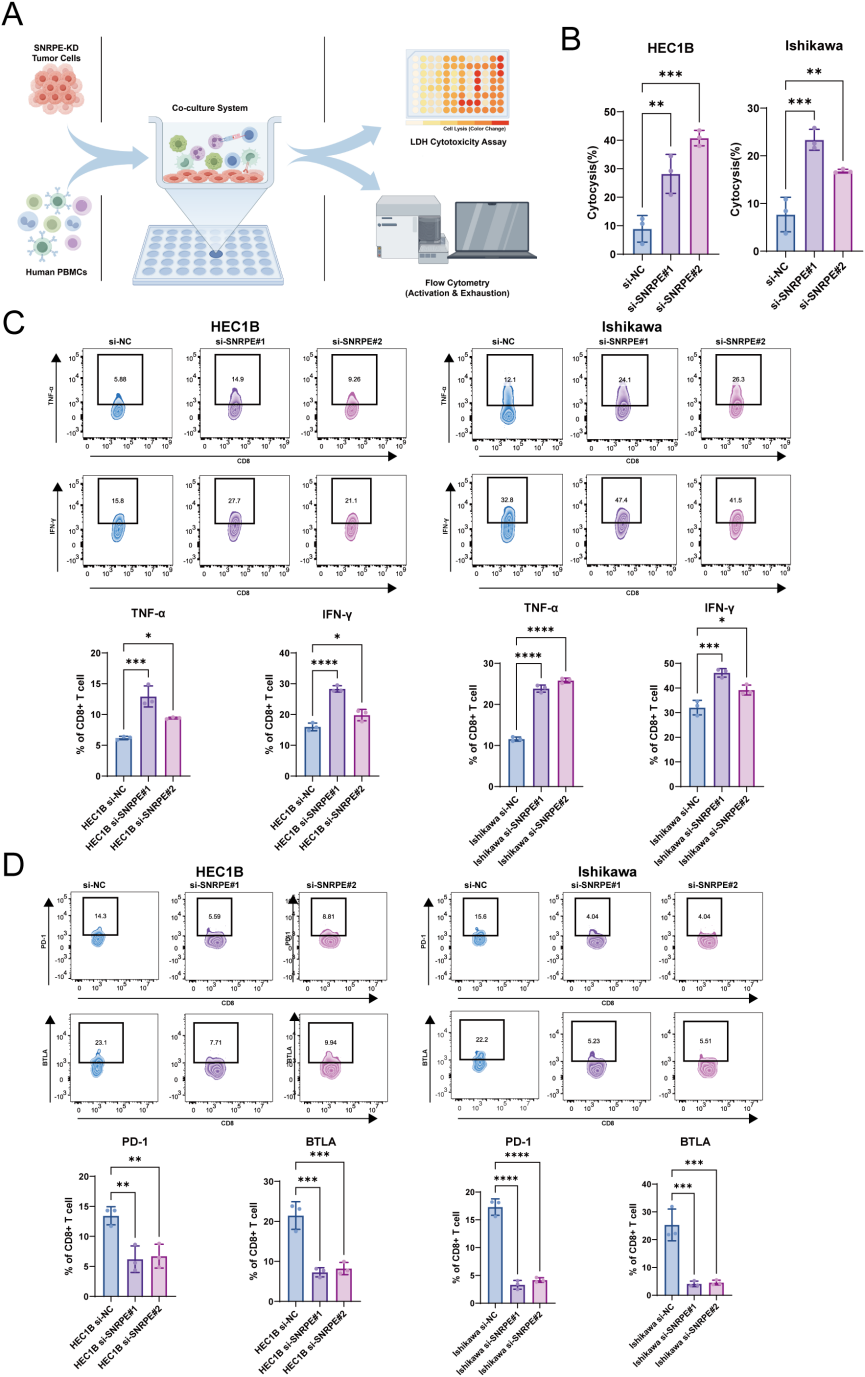
SNRPE depletion restores CD8^+^ T-cell cytotoxicity and reverses exhaustion phenotypes *in vitro*. **(A)** Schematic illustration of the co-culture system. By Figdraw. **(B)** LDH cytotoxicity assay evaluating the killing efficiency of PBMCs against HEC-1B (top) and Ishikawa (bottom) cells transfected with si-NC or si-SNRPE. **(C)** Flow cytometry analysis of CD8^+^ T-cell activation. Representative flow cytometry plots (top) and statistical quantification (bottom) of TNF-α^+^ and IFN-γ^+^ CD8^+^ T cells. **(D)** Flow cytometry analysis of CD8^+^ T-cell exhaustion. Representative plots (top) and quantification (bottom) of PD-1^+^ and BTLA^+^ CD8^+^ T cells. *p < 0.05, **p < 0.01, ***p < 0.001, ****p < 0.0001.

To validate these findings, an *in vitro* co-culture model of SNRPE-knockdown tumor cells with human PBMCs was established (**Figure 8A**). LDH cytotoxicity assays revealed that downregulation of SNRPE significantly enhanced CD8^+^ T cell-mediated killing of tumor cells (**Figure 8B**). Flow cytometry further showed that after co-culture with SNRPE-knockdown tumor cells, CD8^+^ T cells exhibited restored function, with increased activation markers (tumor necrosis factor-α (TNF-α) and interferon-γ (IFN-γ)) and decreased exhaustion markers (programmed cell death protein 1 (PD-1) and B and T lymphocyte attenuator (BTLA)) (**Figures 8C, D**). Collectively, these findings validate the bioinformatics predictions and demonstrate that SNRPE acts as an essential effector of immune evasion in UCEC. Specifically, our *in vitro* data indicate that SNRPE facilitates tumor escape by impairing CD8^+^ T cell-mediated cytotoxicity and driving T cell exhaustion.

### 3.5 SNRPE extensively remodels the transcriptomic landscape and splicing networks in UCEC cells

To elucidate the molecular mechanisms underlying SNRPE-mediated tumor progression, we performed RNA-seq on SNRPE-knockdown HEC-1B cells. The transcriptomic profiling revealed distinct gene expression patterns (**Supplementary Figure 5A**). Functional enrichment analysis of the DEGs demonstrated a significant downregulation of proliferation-related pathways, including the cell cycle, DNA replication, and mitosis (**Supplementary Figure 5B**). This finding is highly consistent with the growth-suppressive phenotypes observed *in vitro* and vivo assays. To validate the RNA-seq data, the transcriptional alterations of six key cell cycle-related genes were experimentally verified via qRT-PCR, yielding results highly concordant with the sequencing heatmap (**Supplementary Figures 5C, D**). After establishing the robustness of our RNA-seq data regarding transcript abundance, we next explored the global impact of SNRPE on post-transcriptional regulation, with a particular focus on AS.

**Figure 9:**
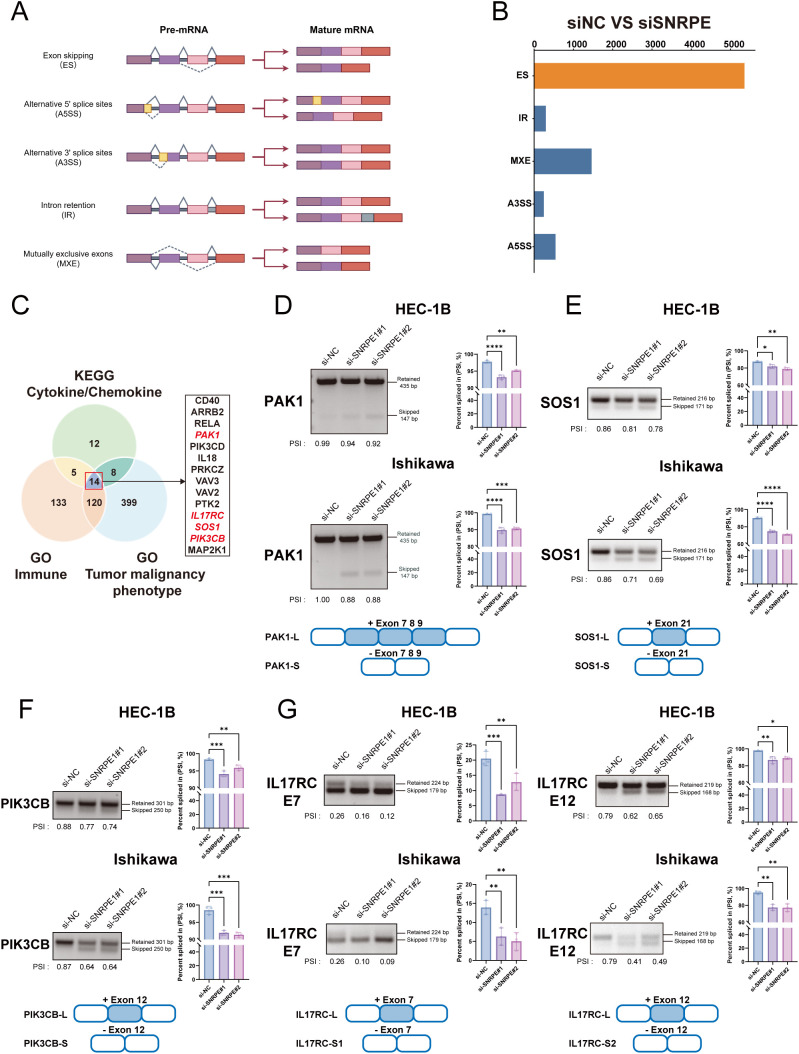
Identification and validation of SNRPE-mediated ES events coordinating tumor-intrinsic and extrinsic pathways. **(A)** Schematic illustration of the 5 basic modes of eukaryotic pre-mRNA AS. By Figdraw. **(B)** global landscape of SNRPE-regulated AS events in UCEC cells. **(C)** Multi-dimensional intersection analysis for the identification of core candidate target genes. **(D–G)** Experimental validation of ES in PAK1 **(D)**, SOS1 **(E)**, PIK3CB **(F)**, and IL17RC **(G)** following SNRPE knockdown. Left panels: representative agarose gel electrophoresis of RT-PCR products using flanking primers, showing the shift in ratio between exon retained and skipped isoforms. Right panels: qPCR analysis of splicing isoforms using sequence-specific junction-spanning primers. *p < 0.05, **p < 0.01, ***p < 0.001, ****p < 0.0001.

In eukaryotes, AS predominantly encompasses 5 basic patterns: exon skipping (ES), intron retention (IR), alternative 5’ splice sites (A5SS), alternative 3’ splice sites (A3SS), and mutually exclusive exons (MXE) (**Figure 9A**). Given that the spliceosome constitutes the core machinery for RNA splicing, we next investigated the genome-wide impact of SNRPE on AS regulation. Our analysis revealed that ES was the most prevalent AS event responsive to SNRPE knockdown, followed by other splicing patterns (**Figure 9B**). Applying stringent filtering criteria (|ΔPSI| > 0.1 and FDR < 0.05), we identified a total of 2,881 target genes exhibiting significant splicing alterations. Together, these results demonstrate that beyond influencing transcript abundance, SNRPE acts as a master regulator of RNA splicing in UCEC cells, where its dysregulation broadly reshapes the cellular transcriptomic landscape.

Given the dual impact of SNRPE knockdown on tumor-intrinsic malignant behaviors and CD8^+^ T cell function, we hypothesized that SNRPE might regulate both biological dimensions by regulating a specific subset of key genes. Thus, we performed comprehensive GO and KEGG enrichment analyzes on the targets exhibiting significant splicing alterations. We extracted genes closely associated with immune responses and cytokine/chemokine signaling pathways. Subsequently, this immune-related cohort was intersected with gene sets known to drive tumor malignant phenotypes. Through this rigorous multidimensional screening, we ultimately identified 14 core candidate target genes (**Figure 9C**). Theoretically, these genes serve as critical “bridges” linking tumor-intrinsic malignancy with the remodeling of the extrinsic immune microenvironment.

To identify the specific downstream targets regulated by SNRPE, we employed two complementary experimental approaches to assess the 14 core target genes in SNRPE-knockdown cells. First, we designed flanking primers targeting adjacent constitutive exons to visually evaluate the relative proportions of the exon-retained and exon-skipped isoforms via RT-PCR coupled with agarose gel electrophoresis. Subsequently, we utilized highly specific junction-spanning primers in qRT-PCR assays to precisely quantify the abundance of both splice variants. SNRPE knockdown effectively shifted the splicing profiles of four key genes: PAK1, SOS1, PIK3CB, and IL17RC. Specifically, these mutually corroborating assays consistently demonstrated that SNRPE knockdown markedly promoted specific exon skipping events within these transcripts (**Figures 9D–G**).

**Figure 10:**
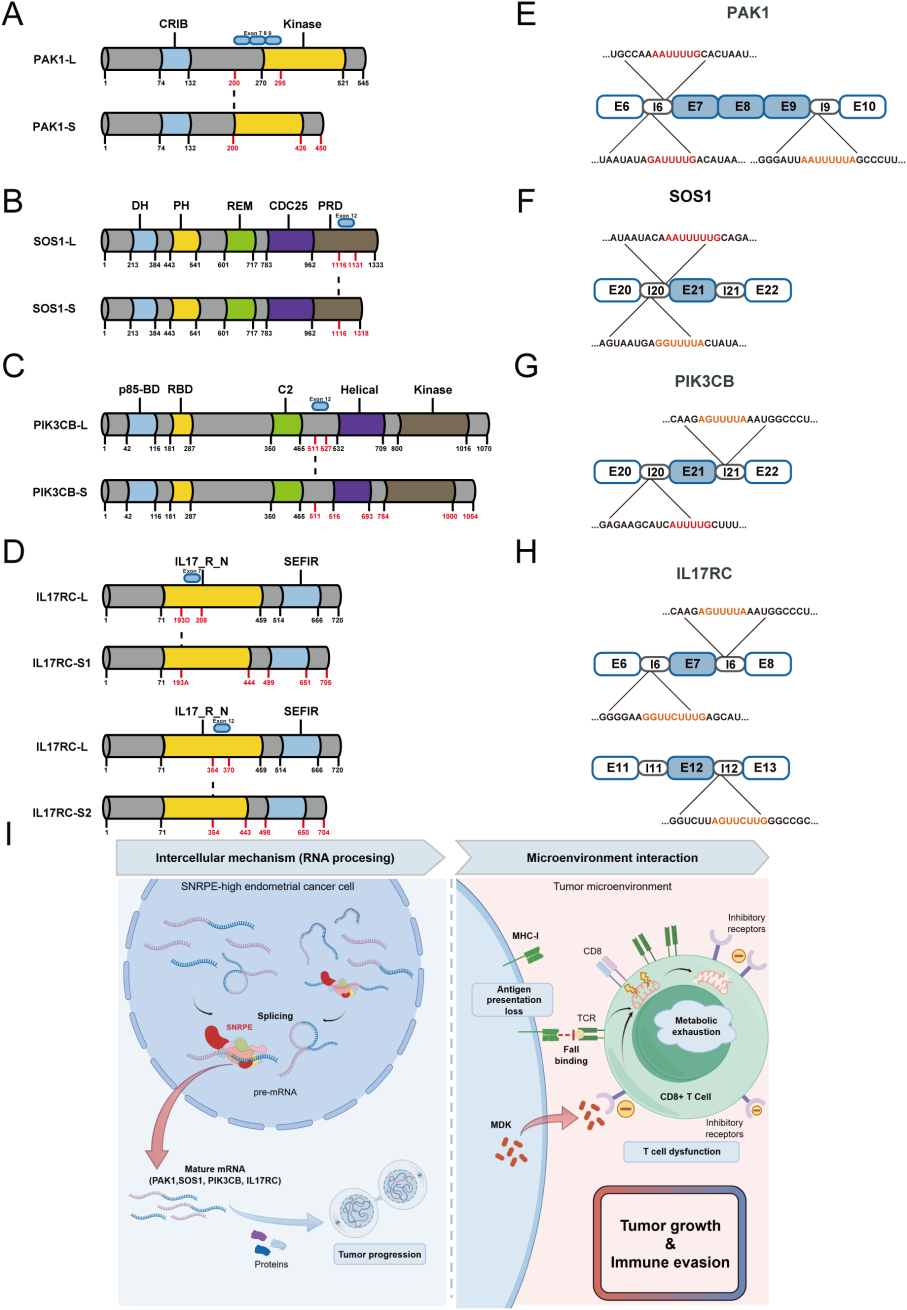
Computational prediction of SNRPE binding motifs and the subsequent impact on protein domain architectures. Schematic representation of alterations in protein domain architectures of PAK1 **(A)**, SOS1 **(B)**, PIK3CB **(C)**, and IL17RC **(D)** induced by SNRPE knockdown. **(E–H)** Predicted SNRPE binding motifs within the critical splice junctions of target pre-mRNAs identified via RBPmap. Conserved sites fully matching the canonical U-rich sequence (RAU_4-6_G) are highlighted in red, while physiological variants with potential binding affinity are indicated in orange. **(I)** A proposed working model of SNRPE-mediated UCEC progression and microenvironment remodeling. By Figdraw. SNRPE functions as a post-transcriptional hub that regulates the splicing pattern of dual-function targets, thereby concurrently sustaining malignant proliferation and immune evasion.

At the structural level, the aberrant splicing induced by SNRPE knockdown directly dismantles the core functional domains of these targets, perfectly mirroring our dual-dimensional screening rationale (**Figures 10A–C**). For critical signaling hubs driving tumor progression, ES resulted in severe structural defects. Specifically, the splicing variant of PAK1 directly deleted the core segment of its kinase catalytic domain, rendering the protein completely kinase-dead. For SOS1, the loss of the critical motif within its C-terminal proline-rich domain (PRD) abolished its interaction with the adaptor protein Grb2. This spatial decoupling traps the protein in the cytosol, preventing its anchorage to the plasma membrane to activate Ras. Meanwhile, PIK3CB lost its essential inter-domain flexible linker, trapping the protein in a state of conformational constraint and thereby preventing its allosteric activation. By altering these key structural motifs, SNRPE-mediated AS effectively shuts down the MAPK and PI3K/AKT signaling cascades (42–44). Crucially, hyperactivation of these specific cascades is well-documented not only to drive autonomous cell cycle progression but also to actively suppress MHC-I antigen presentation and promote the secretion of immunosuppressive factors (45–47). Thus, by structurally disabling these signaling hubs, SNRPE depletion concurrently halts intrinsic tumor growth and removes the oncogenic signals responsible for CD8^+^ T cell exclusion.

Beyond disrupting autonomous growth signals, these structural changes similarly impair the mechanisms of tumor-microenvironment communication. For instance, IL17RC, a critical node in this crosstalk, undergoes targeted skipping of exons 7 or 12 upon SNRPE depletion. This splicing shift yields a truncated isoform lacking the N-terminal extracellular ligand-binding domain (IL17_R_N), thereby disrupting the receptor-ligand interactions necessary for TME communication (**Figure 10D**). This structural defect essentially “blinds” the tumor cells to external IL-17 cytokines, silencing the IL17RC signaling axis (48). Given that IL-17 signaling robustly stimulates tumor cell proliferation, survival, and invasive capacity through downstream inflammatory cascades, the loss of this functional receptor directly attenuates the intrinsic malignant phenotype of UCEC cells (49). Concurrently, because tumor-intrinsic IL-17 signaling heavily dictates the recruitment of immunosuppressive cells and the exclusion of cytotoxic T lymphocytes, this splicing-induced truncation severs a critical communication line, reprogramming the TME from a “cold” to an immune-permissive state.

Collectively, these targets validate our initial screening strategy, highlighting a functional intersection within the tumor ecosystem. In UCEC, SNRPE acts as a key regulatory factor that sustains both cell-intrinsic malignancy and extrinsic immune evasion by ensuring the proper splicing and structural integrity of these critical hubs. Specifically, SNRPE preserves the functional isoforms of signaling nodes (PAK1, SOS1, PIK3CB) to drive autonomous growth, while maintaining the ligand-sensing capacity of IL17RC to facilitate immune escape. Our findings demonstrate that the knockdown of SNRPE collapses these interconnected networks, providing a robust mechanistic foundation for its dual-oncogenic role.

Having established the regulatory role of SNRPE in the AS of these target genes, we sought to determine whether it exerts its function through direct pre-mRNA binding. Therefore, we extracted the sequences of the skipped exons along with their 200-bp upstream and downstream flanking intronic regions, and subjected them to motif prediction using the RBPmap tool. By applying a cross-species conservation filter, we successfully identified highly conserved, canonical Sm protein-binding motifs at the critical splice boundaries of the target exons. Specifically, conserved sites perfectly matching the classical uridine-rich sequence (RAU_4-6_G) were highlighted in red, whereas physiological variants with potential binding capacity (comprising GGUUUUA, AAUUUUUA, AGUUUUA, GGUUCUUUG, and AGUUCUUG) are indicated in orange (50) (**Figures 10E–H**). This evolutionarily conserved sequence evidence strongly indicates that under physiological conditions, SNRPE ensures the precise retention of these core exons by directly anchoring to critical boundary sites on the target pre-mRNAs. In contrast, the depletion of SNRPE prevents its recruitment to these target splice sites, thereby compromising the assembly of the spliceosome, which would lead to aberrant exon skipping, which ultimately yields truncated protein isoforms that can no longer sustain their oncogenic or immunosuppressive functions.

Based on these comprehensive findings, we propose a global working model illustrating SNRPE-mediated endometrial cancer progression and immune microenvironment remodeling (**Figure 10I**). Briefly, acting as a core post-transcriptional regulatory hub, SNRPE orchestrates precise splicing reprogramming to employ a two-pronged mechanism: simultaneously sustaining the intrinsic oncogenic proliferation engine and the extrinsic immune evasion network. Consequently, targeting SNRPE comprehensively dismantles this highly intricate pro-tumorigenic network.

### 3.6 Correlation of SNRPE expression with Ki67 index and CD8+ T cell proportion in clinical UCEC cohorts

**Figure 11:**
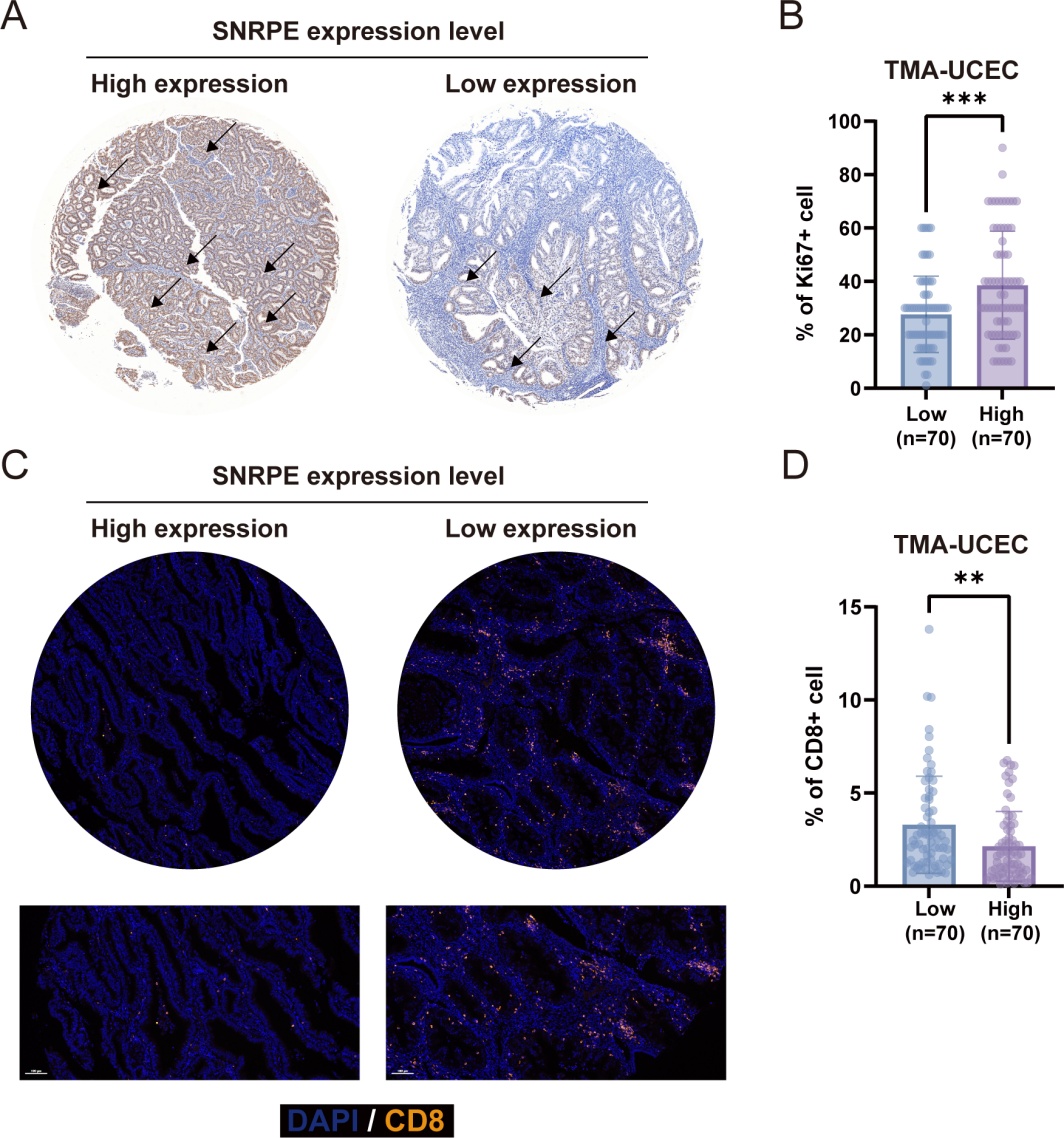
Clinical validation of the correlation between SNRPE expression, tumor proliferation, and CD8^+^ T cell proportion in a UCEC cohort. **(A)** Representative IHC images showing high and low SNRPE expression in the UCEC TMA. Black arrows indicate tumor parenchymal cells. **(B)** Quantitative comparison of the Ki67^+^ cell percentage between the SNRPE-low and SNRPE-high groups (n = 70 per group). **(C)** Representative IF images of serial TMA sections illustrating CD8^+^ cell (orange). Nuclei were counterstained with DAPI (blue). **(D)** Quantitative analysis of the CD8^+^ cell percentage between the two groups. **p < 0.01, ***p < 0.001.

To validate the clinical relevance of SNRPE in tumor progression and the immune microenvironment, we analyzed a TMA cohort of 140 UCEC patients. Based on the IHC staining intensity of SNRPE, the patients were stratified into SNRPE-high (n = 70) and SNRPE-low (n = 70) expression groups (**Figure 11A**). The baseline clinicopathological characteristics, including histological grade, myometrial invasion, lymphovascular space invasion, and clinical stage, were well-balanced and comparable between the two groups (**Supplementary Table 2**). Within this balanced cohort, we retrospectively retrieved the Ki67 proliferation index from the patients’ official diagnostic pathology reports. The percentage of Ki67^+^ cells was significantly higher in the SNRPE-high group than in the SNRPE-low group (**Figure 11B**). This clinical observation corroborates our *in vitro* and vivo findings that SNRPE promotes tumor proliferation.

To next evaluate the effect of SNRPE on intratumoral T cell proportion, we performed IF staining of CD8 on adjacent serial sections from the same TMA cohort. Quantitative analysis revealed a significant reduction in the percentage of CD8^+^ cells within SNRPE-high tumors compared to SNRPE-low tumors (**Figures 11C, D**). These tissue-level data support our bioinformatic prediction that elevated SNRPE expression is linked to an immunosuppressive TME characterized by decreased CD8^+^ T cell proportion.

## 4 Discussion

By integrating GWAS, sQTL, and eQTL data (19–21), SNRPE dysregulation was linked directly to host genetic variants, establishing it as an early oncogenic event rather than a secondary consequence of tumor progression. Cross-comparison with core splicing factor networks further positioned SNRPE as an upstream regulatory hub (22). Clinically, consistent with its oncogenic roles in hepatocellular, breast, and prostate cancers (23–26), SNRPE independently predicted poor PFS and DFS in UCEC patients, suggesting its potential utility for enhanced risk stratification beyond traditional clinical models.

Functional studies *in vitro* and *in vivo* confirmed the essential oncogenic function of SNRPE. Knockdown of SNRPE markedly suppressed UCEC proliferation. Importantly, SNRPE also remodeled the TME to drive immune evasion. ScRNA-seq and multiple deconvolution algorithms revealed a dual immune suppression mechanism. High SNRPE expression reduced MHC-I complex assembly, eliminating tumor immunogenicity and preventing CD8^+^ T cell recognition (51–53).Concurrently, SNRPE upregulated the inhibitory ligand MDK (54, 55), forming a highly specific SNRPE-MDK immunosuppressive axis in UCEC. This hostile TME imposed metabolic stress on CD8^+^ T cells, forcing them into a compensatory “high glycolysis” state that accelerates exhaustion rather than enhancing cytotoxic function (56, 57). *In vitro* PBMC co-culture experiments validated these findings, showing that SNRPE knockdown restored T cell cytotoxicity and reversed exhaustion phenotypes.

Mechanistically, our evidence demonstrated that SNRPE exerts highly selective control over AS, transcending its constitutive role as a basal spliceosomal component. By physically recognizing conserved Sm-binding motifs on pre-mRNAs, SNRPE regulated the splicing pattern of a cohort of pivotal hub genes, including PAK1, SOS1, PIK3CB, and IL17RC. We found that SNRPE deficiency induces targeted ES events that manifest as a spectrum of critical structural defects, including the catalytic inactivation of PAK1 (kinase-dead state), the conformational locking of PIK3CB, the impaired membrane anchorage of SOS1 due to PRD disruption, and the precise truncation of the IL17RC extracellular domain. Crucially, the biological impact of these structural impairments was two-pronged. They not only abrogated cell-intrinsic signaling cascades driving tumor progression but also disrupted the extrinsic immune-regulatory crosstalk within the microenvironment. This multi-dimensional disruption at the protein level provided a rational basis for the observed synergy between growth attenuation and immune sensitization, establishing SNRPE as the mechanistic nexus for the dual-targeting intervention proposed in this study.

Consistent with bioinformatics analysis and *in vitro* and *in vivo* experimental results, clinical UCEC cohorts also reflected the dual impact of SNRPE. In a patient-derived TMA, tumors with high SNRPE expression exhibited a significantly higher Ki67 index and reduced CD8^+^ T cell density. This clinical validation provided crucial tissue-level evidence that the association between SNRPE, accelerated tumor growth, and an immunosuppressed microenvironment extends to human patients.

Despite these insights, limitations exist. First, GWAS-integrated signals were filtered using a suggestive threshold (*p* < 0.05) rather than conventional genome-wide significance, but focusing on functionally validated eQTL/sQTL regions improved sensitivity and reduced false positives. Second, while single-cell analysis showed dual signatures of high SNRPE expression (MHC-I downregulation and MDK upregulation) TCGA validation confirmed only the MDK correlation, suggesting MHC-I suppression may be indirect via secondary signaling or post-transcriptional modifications. Third, while our sequence-based analysis identified potential SNRPE binding sites at splice junctions of downstream target pre-mRNAs, direct experimental confirmation of this RNA–protein interaction remains absent. Fourth, the lack of a fully functional immune system in the xenograft model limits our ability to evaluate the impact of SNRPE on antitumor immunity *in vivo*. Finally, while TMAs and scRNA-seq analyzes confirmed a reduction in CD8^+^ T-cell density in SNRPE-high tumor microenvironments, they lack the spatial resolution to discriminate between tumor stroma and parenchyma.

To address these limitations, future investigations will focus on three main directions. First, RNA immunoprecipitation (RIP) or CLIP assays will be performed to validate the binding between SNRPE and the pre-mRNAs of downstream target genes. Second, to overcome the immunological constraints of xenograft models, subsequent studies will employ immunocompetent transgenic or syngeneic mouse models and explore the potential synergy between SNRPE-targeted therapy and anti-PD-1 treatment, thereby advancing clinical translation. Ultimately, spatial transcriptomics or region-specific imaging techniques will be integrated to acquire the high-resolution spatial information required to distinguish tumor stroma from parenchyma and to determine whether SNRPE influences CD8^+^ T-cell infiltration.

## Data Availability

The original contributions presented in the study are included in the article/**Supplementary Material**. Further inquiries can be directed to the corresponding author.
