# Supplementary material for: Integrative multi-omics analysis identifies SNRPE as a key driver gene in uterine corpus endometrial carcinoma: promoting tumor progression, and mediating immune evasion
Source: Front Immunol. 2026 May 21;17:1827474. doi: 10.3389/fimmu.2026.1827474 (PMC13233447; doi:10.3389/fimmu.2026.1827474)
Supplement: Supplementary file 1 [file DataSheet1.docx]

# Supplementary Figures


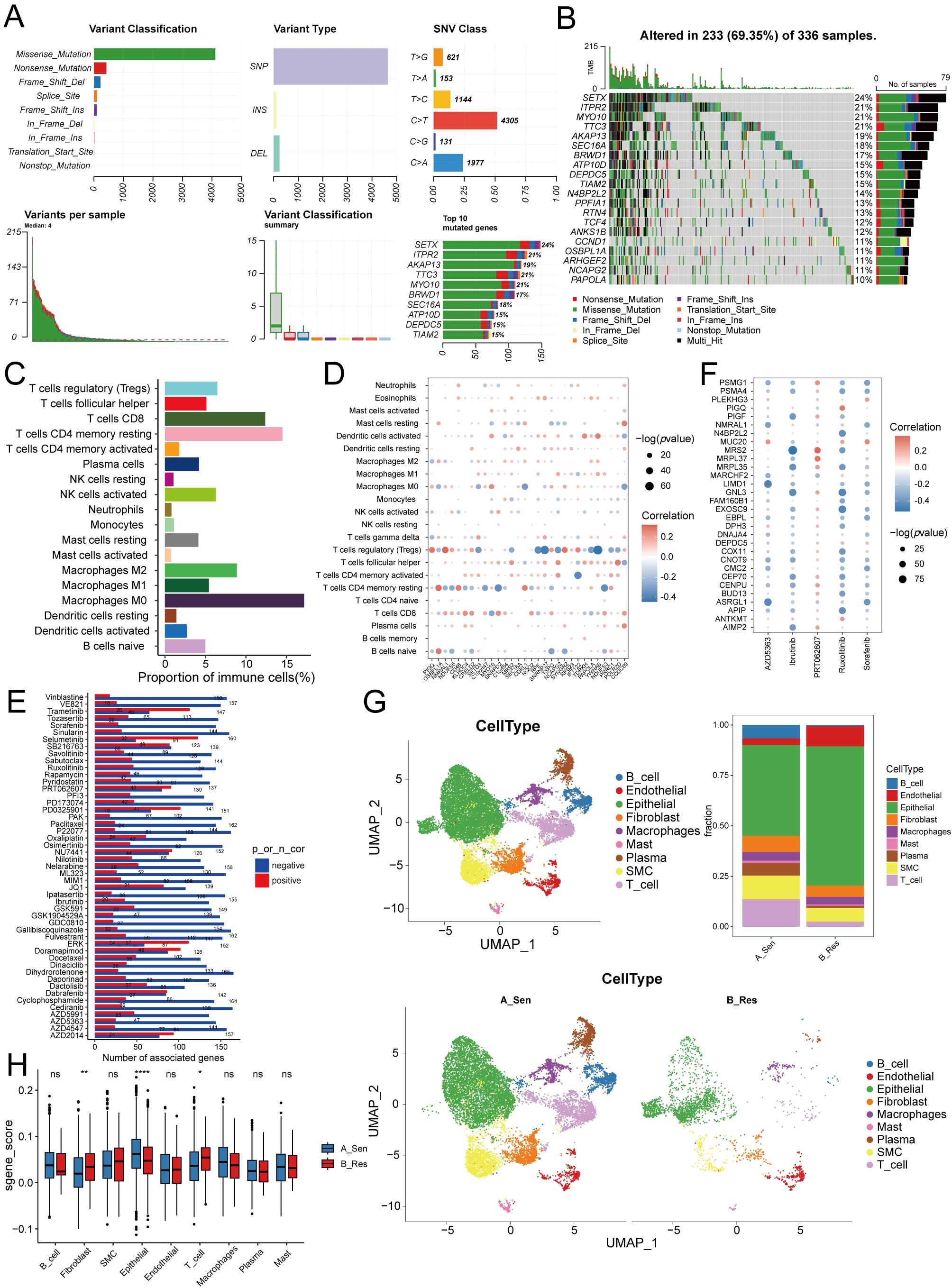


**Supplementary Figure 1** Landscape of genomic alterations, immune microenvironment, and pharmacological profiles of sGenes in UCEC. (A) Summary of somatic mutation profiles of sGenes in UCEC. (B) Oncoplot displaying the detailed mutation landscape of sGenes. (C) Relative proportions of 22 infiltrating immune cell subtypes in UCEC tumor samples (CIBERSORT). (D) Bubble plot visualizing the correlations between sGene expression and immune cell infiltration levels. (E) Pharmacogenomic analysis linking sGenes to drug sensitivity (GDSC2 database). (F) Dot plot showing correlations between the top 30 sGenes and RTK signaling-targeted drugs. (G) Single-cell landscape of the GSE251923 dataset: UMAP visualization (top left), cell type proportions (top right), and UMAP projections stratified by treatment response (A_Sen: Sensitive; B_Res: Resistant). (H) Boxplot comparing the sGene signature scores across different cell types between Sensitive (A_Sen) and Resistant (B_Res) groups.


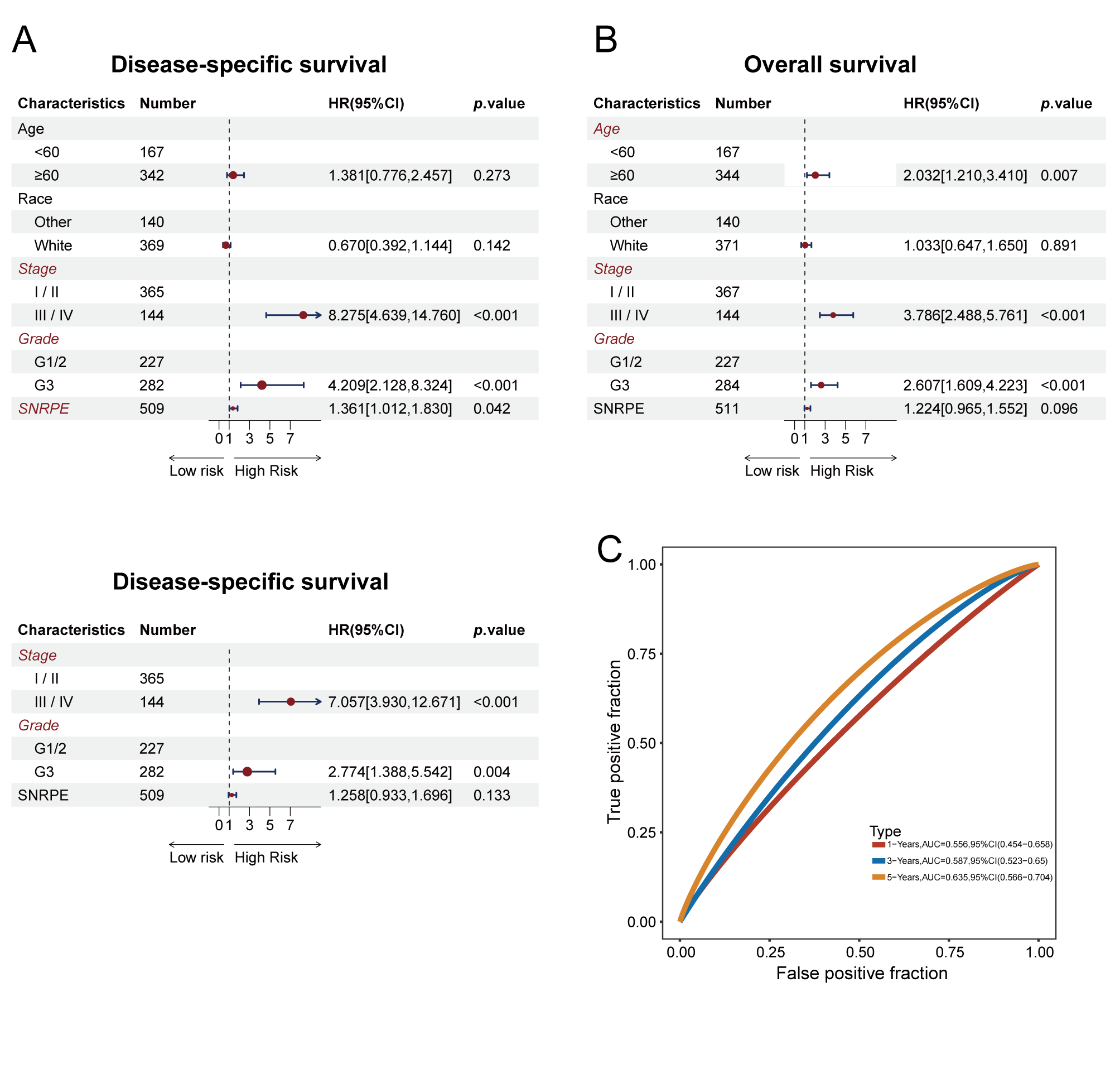


**Supplementary Figure 2** Survival analysis of SNRPE for additional endpoints and predictive accuracy. (A) Forest plots of univariate and multivariate Cox regression analysis for DSS. (B) Forest plot of univariate Cox regression analysis for OS. (C) Time-dependent ROC curves evaluating the predictive accuracy of SNRPE expression for OS at 1, 3, and 5 years.


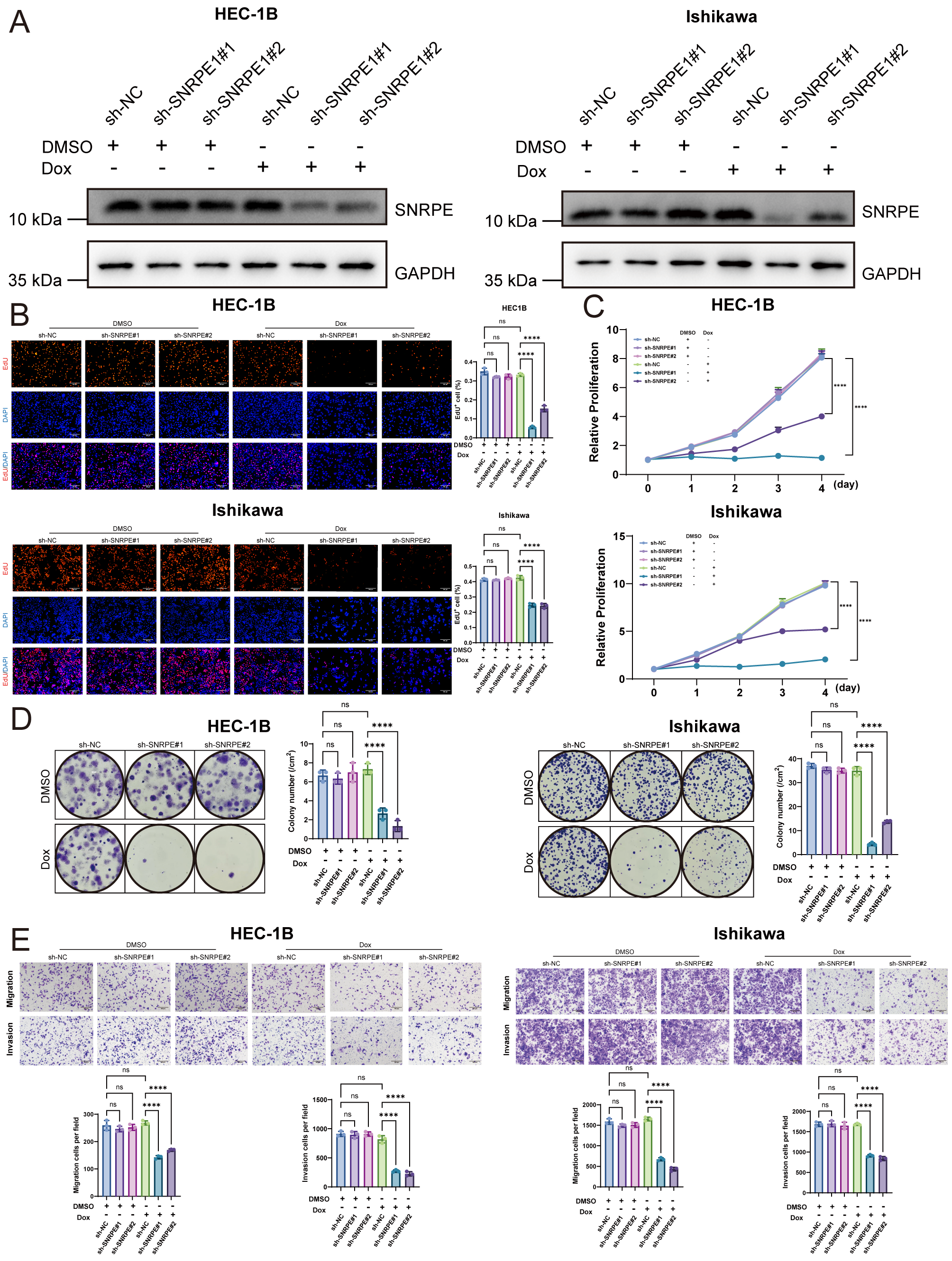


**Supplementary Figure 3**In vitro validation of the Dox-inducible (Tet-on) SNRPE knockdown system. (A) Western blot analysis confirming SNRPE silencing efficiency in HEC-1B and Ishikawa Tet-on cells after Dox treatment. (B) CCK-8 assays showing reduced viability of both cell lines with Dox. (C) EdU incorporation assays indicating decreased DNA synthesis upon Dox induction. (D) Colony formation assays showing reduced clonogenic ability. (E) Transwell migration and invasion assays of HEC-1B and Ishikawa Tet-on cells treated with or without Dox.


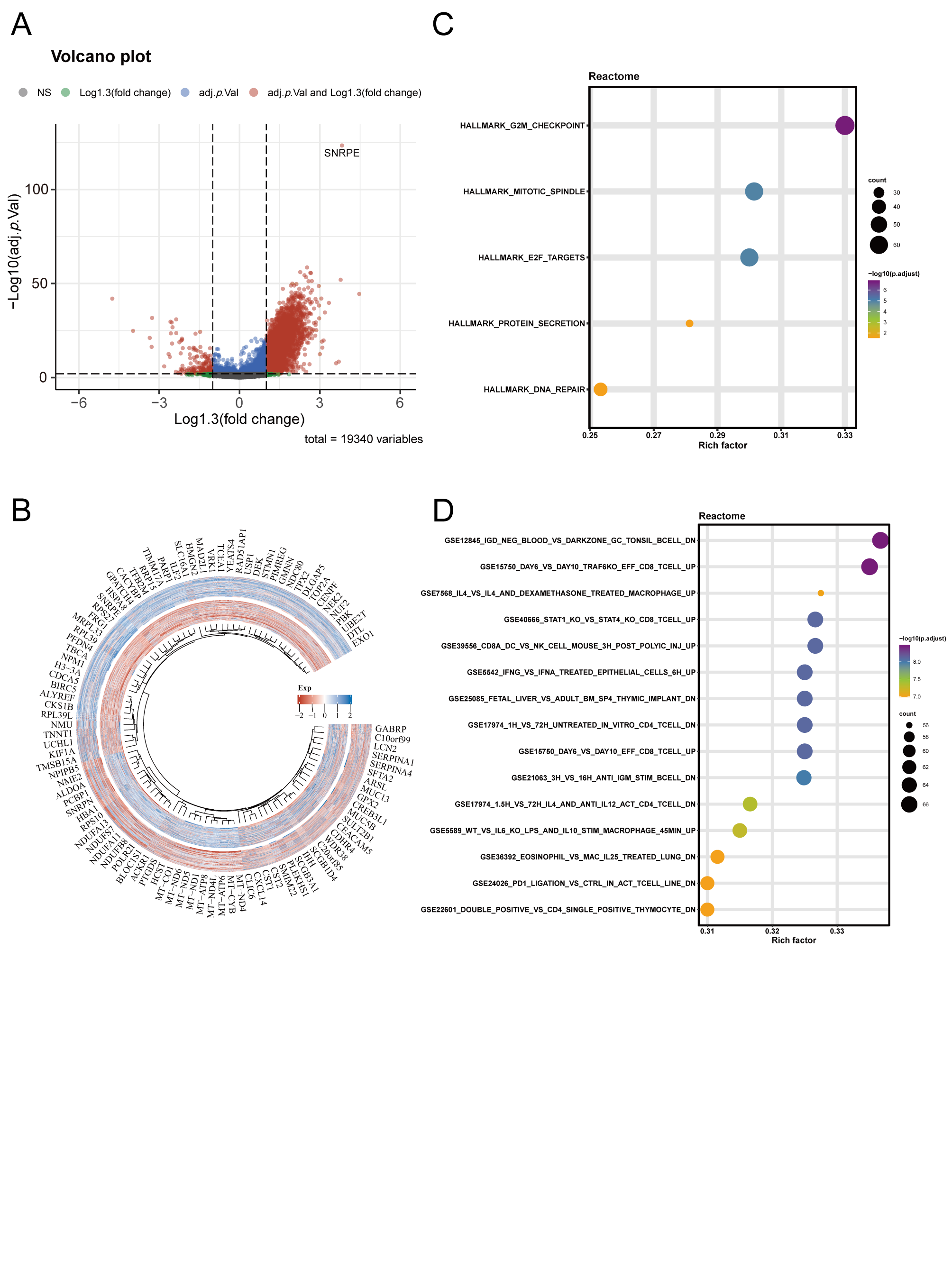


**Supplementary Figure 4** Transcriptomic landscape of SNRPE in clinical samples and additional functional annotations of splicing targets. (A-B) Transcriptomic analysis of the TCGA-UCEC cohort. (A) Volcano plot of DEGs between SNRPE-high and -low groups. (B) Heatmap of top 50 upregulated and downregulated DEGs. (C-D) Functional enrichment of AS genes in HEC-1B cells. (C) Hallmark pathway enrichment analysis. (D) Immune pathway enrichment analysis (MSigDB C7).


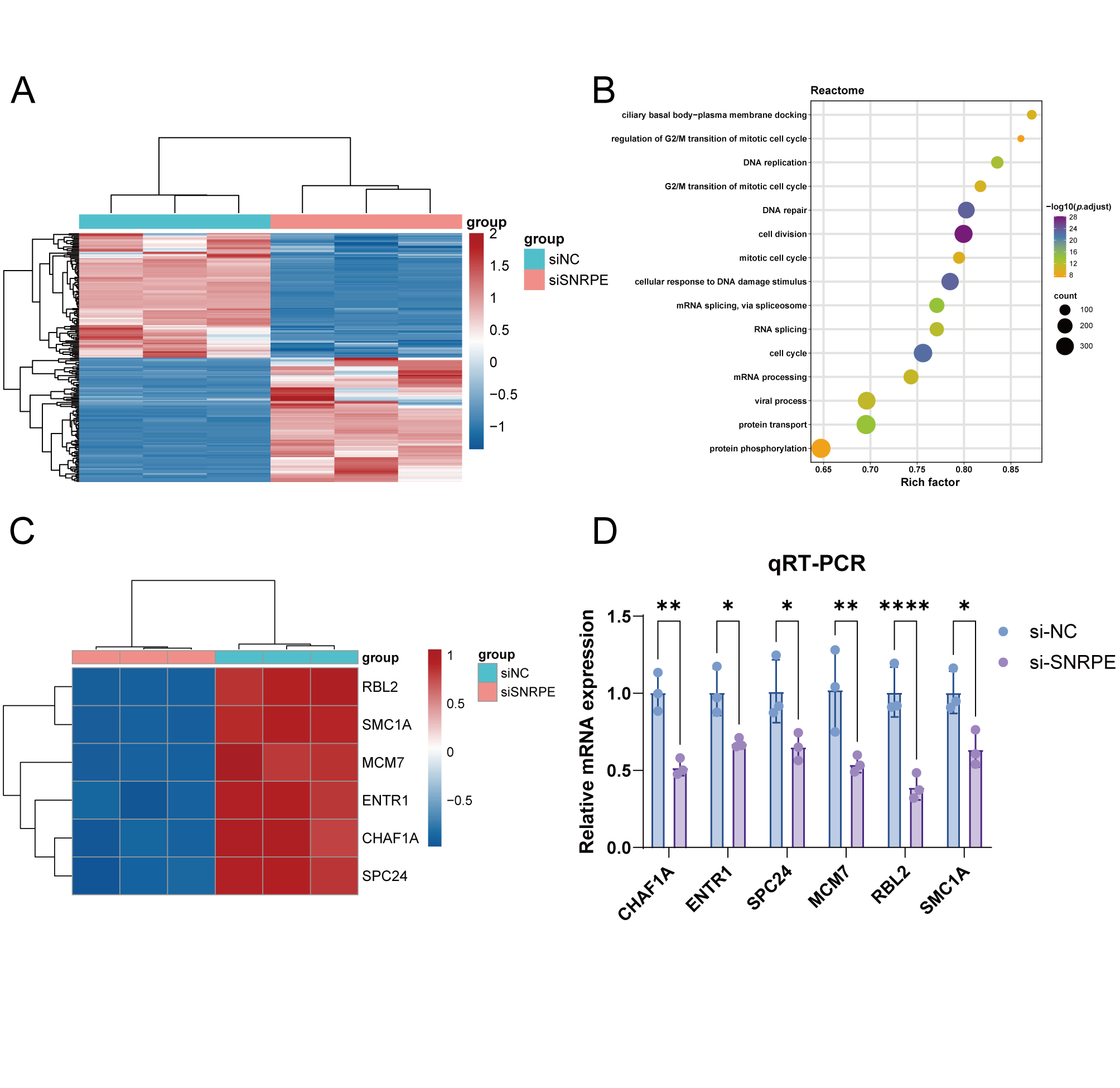


**Supplementary Figure 5** Transcriptomic profiling reveals that SNRPE depletion suppresses cell cycle-related pathways in UCEC cells. (A) Global transcriptomic profile. Heatmap showing the hierarchical clustering of DEGs between si-NC and si-SNRPE groups. (B) Functional enrichment of DEGs. GO enrichment bubble plot of downregulated genes. (C) Heatmap illustrating the relative expression patterns of representative DEGs, specifically focusing on key cell cycle-related genes across the different treatment groups. (D) Experimental validation of the RNA-seq data via qRT-PCR. The relative mRNA expression levels of 6 key cell cycle-related genes were quantified in HEC-1B cells following SNRPE silencing.

# Supplementary Tables (Supplementary Table 4 is provided as an Excel file.)

| **Gene** | **Sequence** |
| --- | --- |
| si-SNRPE#1 | GCAGAAGAGAUUCAUUCUATT |
| si-SNRPE#2 | GCGGAUAGAAGGCUGUAUCTT |
| si-SNRPE#3 | GCUACAAAGUGUCUCCAACTT |
| Tet-on-sh-SNRPE#1 | GCAGAAGAGATTCATTCTAAA |
| Tet-on-sh-SNRPE#2 | GCCTCATTCTAAGTGCTATTT |
| Tet-on-sh-SNRPE#3 | GCTACAAAGTGTCTCCAACTA |

**Supplementary Table 1. siRNA and shRNA sequence information.**

| **Gene Symbol** | **Forward Primer (5' →3')** | **Reverse Primer (5' →3')** |
| --- | --- | --- |
| SNRPE | GCAGCCCATCAACCTCATCTT | GCATGATCCGACCCAGTTGTT |
| GAPDH | ACACCCACTCCTCCACCTTTG | TCCACCACCCTGTTGCTGTAG |
| CHAF1A | CTGAAGAGGAAGTCAGCGGG | TTCAGCACCAATCTGGCCG |
| ENTR1 | CACAGGCTTCCATCAAGCAAC | CGACACTGGACTCTTGCGAT |
| SPC24 | TCAAGGAGATTGAGGCGGAT | GCCATGATGGATGCCTTTG |
| MCM7 | AGCTTCAATCGCCCCAGAAA | GATGTTGATGTTGCCCCGGA |
| RBL2 | CCCCTAGTCAATGCTGTCCC | TGCAAAGTTGTTCCTGTCACC |
| SMC1A | TCAGAGCCGAGAGAGGGAAA | TGAATGCCCAAGCGAGTCTT |
| PAK1-E789-RT | CGCCCAGAGCACACAAAATC | TCCCACGAGGTAACTGTCCA |
| SOS1-E21-RT | CTGCTTCTGGTGCTTCCAGT | GGGCAGATTCTGGTCGTCTT |
| PIK3CB-E12-RT | AATGTTGAATCCAATGGGAACTGT | TTGTGGGAAAATCTCTCGGCA |
| IL17RC-E7-RT | AGCCCAGGTACGAGAAGGAA | TTTGTGCCACCGGGGTTT |
| IL17RC-E12-RT | GACTGCAACTGCTGACCCTG | GCAGCTTCTCCGAGCTGT |
| PAK1-Inclusion-qPCR | TCCACCAGATGCTTTGACCC | ATCGCCCACACTCACTATGC |
| PAK1-Skipping-qPCR | GCAGTGCCACCAGTTTCAGA | TCTGCTTAATGGCCACAGATTTT |
| SOS1-Inclusion-qPCR | ACTCAAGCAATGATACCGTCTT | AGGAGGGACAGGCACTTCAT |
| SOS1-Skipping-qPCR | CCTTTTCACTCAAGATCTGCTTCTG | GGAGGGACAGGCACTTCATC |
| PIK3CB-Inclusion-qPCR | TGTTGAATCCAATGGGAACTGT | TCTCAGCTGCCTTTTCAATAATCT |
| PIK3CB-Skipping-qPCR | CCTTCGATAAGAGTCGAGGTGG | TGTGGGAAAATCTCTCGGCA |
| IL17RC-E7-Inclusion-qPCR | GCTGCCTGACTGCAGGG | TTTTTGTGCCACCGGGGTTT |
| IL17RC-E7-Skipping-qPCR | TGCCTGCCCTGCCCT | TTTGTGCCACCGGGGTTTT |
| IL17RC-E12-Inclusion-qPCR | GAGAACGTCACTGTGGACAAGGT | CCTGAACACAGAGGTTAGGGTG |
| IL17RC-E12-Skipping-qPCR | ACTGCAACTGCTGACCCTG | TCTCCGAGCTGTTCACGTCC |

**Supplementary Table 2. Primer sequences used for RT-PCR and qRT-PCR.**

**Supplementary Table 3. Chemicals, recombinant proteins and antibodies.**

| **No** | **Reagent** | **Source** | **Cat. No** | **Dilution / Application** |
| --- | --- | --- | --- | --- |
| 1 | DMEM | Gibco | Cat# 11965092 | / |
| 2 | DMEM/F12 | Servicebio | Cat# G4612 | / |
| 3 | RPMI 1640 | Gibco | Cat# 11875093 | / |
| 4 | Fetal Bovine Serum | BI | Cat# 04-001-1A | / |
| 5 | Penicillin-Streptomycin | Thermo Fisher | Cat# 15140122 | / |
| 6 | Lipofectamine RNAiMAX Reagent | Thermo Fisher | Cat# 31985070 | / |
| 7 | DNA transfection reagent | Neofect Biotechnology | Cat# TF20121201 | / |
| 8 | Lentiviral concentrator kit | Genomeditech | Cat# GM-040801 | / |
| 9 | HistransG A | Genechem | Cat# REVG004 | / |
| 10 | Puromycin | MedChemExpress | Cat# 58-58-2 | / |
| 11 | HiScript III All-in-one RT SuperMix | Vazyme Biotech | Cat# R333 | / |
| 12 | ChamQ Universal SYBR qPCR Master Mix | Vazyme Biotech | Cat# Q711 | / |
| 13 | RNAeasy Animal RNA Isolation Kit with Spin Column | Beyotime Biotechnology | Cat# R0027 | / |
| 14 | PVDF membranes | Millipore | Cat# ISEQ00010 | / |
| 15 | ECL chemiluminescence kit | Epizyme Biotech | Cat# SQ202L-1 | / |
| 16 | CCK-8 reagent | MCE | Cat# Cat# HY-K0301 | / |
| 17 | Click-iT EdU Kit | Beyotime Biotechnology | Cat# C0075 | / |
| 18 | Doxycycline | Aladdin | Cat# D302150 | / |
| 19 | Ficoll-Paque solution | GE Healthcare | Cat# 17-1440-02 | / |
| 20 | Dynabeads Human T-Activator CD3/CD28 | Thermo Fisher | Cat# 11131D | / |
| 21 | Recombinant Human IL-2 | BioLegend | Cat# 589104 | / |
| 22 | LDH Cytotoxicity Assay Kit | Beyotime Biotechnology | Cat# C0016 | / |
| 23 | PMA | Sigma | Cat# P1585 | / |
| 24 | Ionomycin | MCE | Cat# HY-13434 | / |
| 25 | Cytofix/Cytoperm™ Plus Fixation/Permeabilization Solution Kit with BD GolgiStop™ | BD Biosciences | Cat# 554715 | / |
| 26 | Matrigel | Corning | Cat# 356234 | / |
| 27 | EmeraldAmp® PCR Master Mix | Takara Bio | Cat# RR300A | / |
| 28 | GAPDH | proteintech | HRP-60004 | IB: 1:10000 |
| 29 | SNRPE | Thermo Fisher | PA5-96342 | IB: 1:2000 / IHC: 1:200 |
| 30 | Ki67 | proteintech | 27309-1-AP | IHC: 1:500 |
| 31 | Anti-rabbit | proteintech | RGAR001 | IB: 1:2000 |
| 32 | PE/Cy7-anti-human CD107a | BioLegend | 328617 | FCM |
| 33 | PerCP/Cy5.5-anti-human CD45 | BioLegend | 368503 | FCM |
| 34 | AmCyan-anti-human CD3 | BioLegend | 300447 | FCM |
| 35 | APC/Cy7-anti-human CD8 | BioLegend | 344713 | FCM |
| 36 | Fixable Viability Dye eFluor 450 | BioLegend | 65-0863-14 | FCM |
| 37 | FITC-anti-human IFN-γ | BioLegend | 502553 | FCM |
| 38 | PE-anti-human TNF-α | BioLegend | 502908 | FCM |
| 39 | FITC-anti-human CD279 | BioLegend | 621631 | FCM |
| 40 | PE-anti-human CD272 | BioLegend | 344505 | FCM |
| 41 | CD8 alpha (C8/144B) Mouse Monoclonal Antibody | Cell Signaling Technology | 70306 | IF: 1:200 |

IB: Immunoblot；IHC: Immuobiochemistry; FCM: Flow Cytometry; IF: Immunofluorescence.

**Supplementary table 5. Clinical validation of the association between SNRPE expression and clinicopathological features in the institutional UCEC cohort.**

| **Clinical characteristic** | **Low SNRPE expression**  **(n = 70)** | **High SNRPE expression**  **(n = 70)** | ***p* value** | |
| --- | --- | --- | --- | --- |
| Grade |  |  |  |  |
| G1/2 | 66 (94.2%) | 63 (90.0%) | 0.532 |  |
| G3 | 4 (5.7%) | 7 (10.0%) |  |  |
| Myometrial invasion |  |  |  |  |
| < 1/2 | 47 (67.1%) | 39 (55.7%) | 0.224 |  |
| ≥ 1/2 | 23 (32.9%) | 31 (44.3%) |  |  |
| Cervical involvement |  |  |  |  |
| Absent | 58 (82.9%) | 53 (75.7%) | 0.404 |  |
| Present | 12 (17.1%) | 17 (24.3%) |  |  |
| Lymphovascular space invasion |  |  |  |  |
| Absent | 57 (81.4%) | 51 (72.9%) | 0.314 |  |
| Present | 13 (18.6%) | 19 (27.1%) |  |  |
| Lymph node metastasis |  |  |  |  |
| Absent | 64 (91.4%) | 65 (92.9%) | 1.000 |  |
| Present | 6 (8.6%) | 5 (7.1%) |  |  |
| Stage |  |  |  |  |
| IA | 42 (60.0%) | 32 (45.7%) | 0.127 |  |
| ≥ IB | 28 (40.0%) | 38 (54.3%) |  |  |

Patients were dichotomized into High and Low expression groups based on the median IHC H-score of SNRPE. *P* values were calculated using the chi-square test, Fisher's exact test, or Mann–Whitney U test, as appropriate.
